# Long-term trends in the burden of cancer attributable to high body mass index in China from 1990 to 2021

**DOI:** 10.3389/fnut.2025.1606747

**Published:** 2025-05-21

**Authors:** Zhouwei Zhan, Yi Zeng, Shaohua Xu, Hongju Chen, Rui Huang, Wei Lin, Jiami Yu, Xiaojie Wang, Chunkang Yang, Zengqing Guo, Bijuan Chen

**Affiliations:** ^1^Department of Medical Oncology, Clinical Oncology School of Fujian Medical University, Fujian Cancer Hospital, Fuzhou, China; ^2^Department of Gastrointestinal Surgery, Clinical Oncology School of Fujian Medical University, Fujian Cancer Hospital, Fuzhou, China; ^3^Department of Hepatobiliary and Pancreatic Surgery, Clinical Oncology School of Fujian Medical University, Fujian Cancer Hospital, Fuzhou, China; ^4^Department of Gynecology, Clinical Oncology School of Fujian Medical University, Fujian Cancer Hospital, Fuzhou, China; ^5^Digestive Endoscopy Center, Clinical Oncology School of Fujian Medical University, Fujian Cancer Hospital, Fuzhou, China; ^6^Department of Radiation Oncology, Clinical Oncology School of Fujian Medical University, Fujian Cancer Hospital, Fuzhou, China

**Keywords:** high BMI, cancer burden, DALYs, mortality, China, Global Burden of Disease, temporal trends, age-period-cohort analysis

## Abstract

**Background:**

High body mass index (BMI) is a well-established modifiable risk factor for multiple cancer types. However, the long-term trends and demographic patterns of total cancer burden attributable to high BMI in China have not been fully characterized. This study aimed to assess the magnitude, temporal trends, and demographic drivers of BMI-related cancer burden in China from 1990 to 2021.

**Methods:**

Data on total cancer burden attributable to high BMI were obtained from the Global Burden of Disease Study 2021. Key indicators included deaths, disability-adjusted life years (DALYs), years lived with disability (YLDs), and years of life lost (YLLs). Age-standardized rates (ASRs) were estimated by sex and age group. Joinpoint regression and age-period-cohort (APC) analyses were performed to assess trends, and decomposition analysis was used to quantify the contributions of population growth, population aging, and epidemiological changes.

**Results:**

In 2021, high BMI accounted for 58,745 cancer deaths and 1.66 million DALYs in China. Compared with 1990, age-standardized death and DALY rates increased by approximately 1.7-fold and 1.6-fold, respectively. Males had higher age-standardized rates of deaths, DALYs, and YLLs, while females exhibited higher YLD rates. The burden peaked in the 50–69 age group and varied by sex across age strata. Colorectal, liver, kidney, and breast cancers were the largest contributors to the BMI-related cancer burden. APC analysis revealed growing burden across age and period dimensions, with higher DALY and mortality rates observed in more recent birth cohorts. Decomposition analysis showed that the increase in deaths was mainly driven by epidemiological change and population growth, while DALY increases were influenced by all three components, with a stronger impact in males.

**Conclusion:**

The burden of cancer attributable to high BMI in China has risen substantially over the past three decades, with clear sex, age, and cohort-specific differences. Comprehensive public health strategies targeting obesity prevention and cancer control are urgently needed.

## Introduction

Over the past few decades, the prevalence of overweight and obesity has risen dramatically worldwide, emerging as a major public health concern (1, 2). In China, the shift in dietary patterns, increased urbanization, and sedentary lifestyles have led to a rapid escalation in high body mass index (BMI) among the adult population (3–5). According to estimates from the Global Burden of Disease (GBD) Study, high BMI is now a leading modifiable risk factor for a range of non-communicable diseases, including type 2 diabetes, cardiovascular conditions, and multiple cancers (6–8). While the causal links between high BMI and specific cancers such as colorectal, breast, endometrial, and kidney cancers are well-established (9, 10), comprehensive data on the overall cancer burden attributable to high BMI, particularly within the context of China’s aging population, remain limited. Given the rising prevalence of obesity and the increasing incidence of cancer in China, quantifying this burden is critical to inform public health strategies and guide targeted interventions.

Existing epidemiological evidence suggests that excess body weight influences cancer development through several mechanisms, including chronic inflammation, insulin resistance, altered levels of sex hormones, and dysregulated adipokine signaling (11, 12). Mendelian randomization studies have provided further support for a causal relationship between elevated BMI and site-specific cancers, notably colorectal, pancreatic, and breast cancers (13–15). In addition to increasing incidence, high BMI has been associated with worse cancer prognosis and reduced survival, particularly among individuals with central adiposity and metabolic syndrome (16, 17). Moreover, sex-based and age-specific differences in BMI-associated cancer risk have been documented, emphasizing the need for disaggregated analysis (18). These patterns point to the importance of a nuanced understanding of how high BMI contributes to cancer burden across diverse demographic strata. However, most available studies have focused on single cancer types or select regions, and comprehensive assessments at the national level remain scarce.

To address this gap, we conducted a systematic analysis of the burden of total cancer attributable to high BMI in China from 1990 to 2021, using data from the GBD 2021 study. Our study aimed to quantify the burden in terms of deaths, disability-adjusted life years (DALYs), years of life lost (YLLs), and years lived with disability (YLDs), disaggregated by sex, age, and cancer type. Additionally, we analyzed temporal trends using joinpoint regression, examined underlying drivers through decomposition analysis, and explored age-period-cohort effects to understand generational shifts. By leveraging standardized and comparable data, this study provides updated evidence on the evolving impact of high BMI on cancer burden in China. These findings have important implications for cancer prevention policies, weight control strategies, and long-term public health planning. As the obesity epidemic continues to expand, addressing the cancer burden associated with high BMI is crucial to mitigating the future health and economic costs in an aging Chinese society.

## Methods

### Data source

This study utilized data from the GBD 2021 study, coordinated by the Institute for Health Metrics and Evaluation (IHME). The GBD provides comprehensive, standardized estimates of mortality and morbidity attributable to a wide range of risk factors across 204 countries and territories (6, 19). All data used in this analysis were accessed through the Global Health Data Exchange (GHDx) results tool,^1^ which allows disaggregation by country, year, sex, age group, and cause. The GBD 2021 study employs a rigorous and standardized methodology to estimate disease burden attributable to various risk factors, including high BMI. For mortality estimates, GBD synthesizes data from vital registration systems, cancer registries, and verbal autopsy records, applying statistical models such as Cause of Death Ensemble modeling (CODEm) to adjust for data incompleteness and misclassification (6, 20). Non-fatal outcomes, including prevalence and YLDs, are estimated using DisMod-MR 2.1, a Bayesian meta-regression tool that integrates data from epidemiological studies, national surveys, and hospital records. Risk-attributable burden is calculated by comparing the observed exposure distribution to a theoretical minimum risk exposure level (TMREL), applying a comparative risk assessment framework. This approach quantifies the proportion of disease burden that could be avoided if all individuals had a BMI within the TMREL range. All estimates are generated with 95% uncertainty intervals (UIs), reflecting uncertainty from data sources, model selection, and parameter estimation.

### Definition and estimation

In GBD 2021, high BMI was defined based on age. For adults aged 20 years and older, high BMI was classified as a BMI greater than the TMREL of 20–23 kg/m^2^. This range represents the BMI associated with the lowest risk of death and disability from all causes in large pooled epidemiological studies and serves as a reference point in the comparative risk assessment framework used by GBD (6). For children and adolescents aged 2–19 years, high BMI was defined as overweight or obesity, according to age- and sex-specific thresholds established by the International Obesity Task Force (IOTF) (6). This approach captures both population-wide risk exposure and age-specific vulnerability to elevated BMI across the life course. Total cancer was defined using International Classification of Diseases, 10th Revision (ICD-10) codes C00-C97, encompassing all malignant neoplasms, including non-melanoma skin cancers. Benign, *in situ*, and unspecified neoplasms were excluded. Twelve site-specific cancers with known associations to high BMI were also assessed, including cancers of the colon and rectum, liver, gallbladder and biliary tract, pancreas, kidney, thyroid, breast (female), uterus, ovary, non-Hodgkin lymphoma, multiple myeloma, and leukemia (6). The cancer burden attributable to high BMI was estimated using the comparative risk assessment (CRA) framework employed by GBD. This involves calculating population attributable fractions (PAFs) by integrating the relative risk of cancer associated with BMI levels above the TMREL and the population exposure distribution.

### Descriptive analysis

Attributable burden was quantified for four main metrics: number of deaths, DALYs, YLLs, and YLDs. DALYs represent the sum of YLLs and YLDs, capturing both fatal and non-fatal consequences of disease. Age-standardized rates per 100,000 population were computed using the GBD world standard population to allow comparability over time and between sexes. All estimates were stratified by sex, age group, and cancer type. The all-age and age-standardized burden of total cancer attributable to high BMI was first summarized for males and females in 2021. The sex- and age-specific distributions of deaths, DALYs, YLLs, and YLDs were visualized to identify peak burden groups and to explore sex-specific differences in fatal and non-fatal components. Temporal trends from 1990 to 2021 were described in terms of both absolute numbers and ASRs. Key turning points and shifts in sex-specific burden over time were noted. For site-specific analyses, the cancer types contributing the largest burden in 2021 were identified, and changes in age-standardized rates over time were evaluated to highlight rapidly increasing cancers. In addition, the proportional distributions of deaths, DALYs, YLLs, and YLDs by sex and cancer type were depicted to characterize differences in burden structure. All burden estimates were reported with 95% UIs, which were generated based on 1,000 posterior draws from the GBD modeling framework (6, 19).

### Joinpoint regression analysis

Joinpoint regression analysis was performed to examine temporal trends in the ASRs of deaths, DALYs, YLLs, and YLDs for total cancer attributable to high BMI in China from 1990 to 2021. The Joinpoint Regression Program (version 5.2.0; National Cancer Institute, United States) was used for all analyses. This method allows for the identification of statistically significant changes in trend, referred to as “joinpoints,” where the slope of the regression line changes significantly (21, 22). A maximum of five joinpoints was allowed for each model, and the best-fitting model was selected based on the permutation test and Bayesian Information Criterion. For each segment between joinpoints, the annual percentage change (APC) and corresponding 95% confidence intervals (CIs) were estimated. The average annual percent change (AAPC) over the entire period (1990–2021) was also calculated to summarize the overall trend for each indicator. All analyses were stratified by sex to assess sex-specific differences in temporal patterns. A log-linear model was applied to the ASRs, and statistical significance was determined at a two-sided *p*-value < 0.05. The direction and magnitude of trends, whether increasing, decreasing, or stable, were reported for each indicator and for both sexes.

### Age-period-cohort analysis

To evaluate the independent effects of age, time period, and birth cohort on the temporal trends in DALY and mortality rates of total cancer attributable to high BMI, an APC model was applied. This analytical framework enables the separation of overlapping influences among the three time-related dimensions and provides insights into how each factor contributes to the observed patterns. Due to the inherent collinearity among age, period, and cohort variables, the intrinsic estimator (IE) method was adopted to generate statistically stable and interpretable effect estimates. Mortality and DALY data were categorized into consecutive 5-year age groups (e.g., 20–24, 25–29, …, 90–94, 95+), 5-year periods (e.g., 1992–1996, 1997–2001, …, 2017–2021), and corresponding birth cohorts (e.g., 1902–1907 to 1997–2001) (23, 24). The 2002–2006 period was used as the reference group for estimating period effects, and the 1952–1956 birth cohort served as the reference for cohort effects. Cumulative incidence and mortality rates were standardized to account for variations across age groups and were used as outcome variables in the APC model. The APC analysis was conducted using the Epi package (version 2.46) in R software (version 4.3.1). Model diagnostics, including residual plots and Akaike Information Criterion (AIC) values, were examined to evaluate model fit and ensure robustness.

### Decomposition analysis

A decomposition analysis was conducted to quantify the relative contributions of three main factors (population growth, population aging, and epidemiological changes) to the increase in total cancer deaths and DALYs attributable to high BMI in China between 1990 and 2021. The decomposition was based on a counterfactual scenario approach. First, the total change in the number of deaths and DALYs between 1990 and 2021 was calculated. This change was then partitioned into three components: (1) population growth, representing the increase in burden due to a larger total population; (2) population aging, representing shifts in the age distribution of the population; and (3) epidemiological changes, defined as changes in age-specific mortality or DALY rates, which may reflect changes in exposure to high BMI, healthcare access, diagnostic practices, or treatment outcomes (25). The contributions of each factor were estimated using a stepwise replacement algorithm that isolates each component while holding the others constant. The decomposition was performed separately for males and females to assess sex-specific differences in driving forces behind the burden increase. All calculations were conducted using R software (version 4.3.1). This method provided insight into the extent to which demographic shifts and changing risk profiles have shaped the rising cancer burden attributable to high BMI in China.

## Results

### Burden of total cancer attributable to high BMI in China, 2021

In 2021, high BMI was associated with a notable burden of total cancer in China, as reflected in both mortality and disability metrics (Table 1). A total of 58,745 all-age deaths were attributed to high BMI, with males and females contributing 27,732 and 31,013 deaths, respectively. Despite the higher absolute number of deaths in females, the age-standardized death rate was slightly higher in males (2.87 per 100,000) than in females (2.79 per 100,000), with a combined rate of 2.81 per 100,000 for the total population. The overall DALYs attributable to high BMI reached 1,658,721, with similar sex-specific distributions, though the age-standardized DALY rate was marginally higher in males (81.74 per 100,000) than in females (76.55 per 100,000). YLDs accounted for 71,314 of the DALYs, with females showing a higher age-standardized YLD rate (4.08 per 100,000) compared to males (2.52 per 100,000). In contrast, YLLs, which represented the majority of the burden, totaled 1,587,406 and showed higher age-standardized rates in males (79.22 per 100,000) than in females (72.48 per 100,000), with an overall rate of 75.87 per 100,000. These findings indicate a considerable cancer burden attributable to high BMI in China, with differences in fatal and non-fatal outcomes between sexes.

**Table 1:** All-age cases and age-standardized deaths, DALYs, YLDs, and YLLs rates in 2021 for total cancer attributable to high BMI in China.

Measure	All-ages cases			Age-standardized rates per 100,000 people		
	Total	Male	Female	Total	Male	Female
Deaths	58,745 (24,601, 99,889)	27,732 (12,216, 47,942)	31,013 (12,237, 55,357)	2.81 (1.2, 4.76)	2.87 (1.29, 4.89)	2.79 (1.12, 4.97)
DALYs	1,658,721 (693,186, 2,831,308)	816,759 (364,272, 1,430,644)	841,961 (329,680, 1,507,632)	79.17 (33.82, 134.14)	81.74 (37.41, 141.7)	76.55 (30.7, 136.27)
YLDs	71,314 (25,942, 123,844)	25,739 (11,121, 44,703)	45,575 (14,907, 81,984)	3.3 (1.23, 5.7)	2.52 (1.1, 4.35)	4.08 (1.37, 7.28)
YLLs	1,587,406 (667,393, 2,708,992)	791,021 (353,047, 1,382,532)	796,386 (313,263, 1,423,820)	75.87 (32.6, 128.48)	79.22 (36.14, 137.17)	72.48 (29.26, 128.93)

Values in parentheses indicate 95% UIs, estimated using Monte Carlo simulations. DALYs, disability-adjusted life-years; YLDs, years lived with disability; YLLs, years of life lost; BMI, body mass index; UI, uncertainty interval.

### Age and sex distribution of cancer burden attributable to high BMI in China, 2021

**Figure 1:**
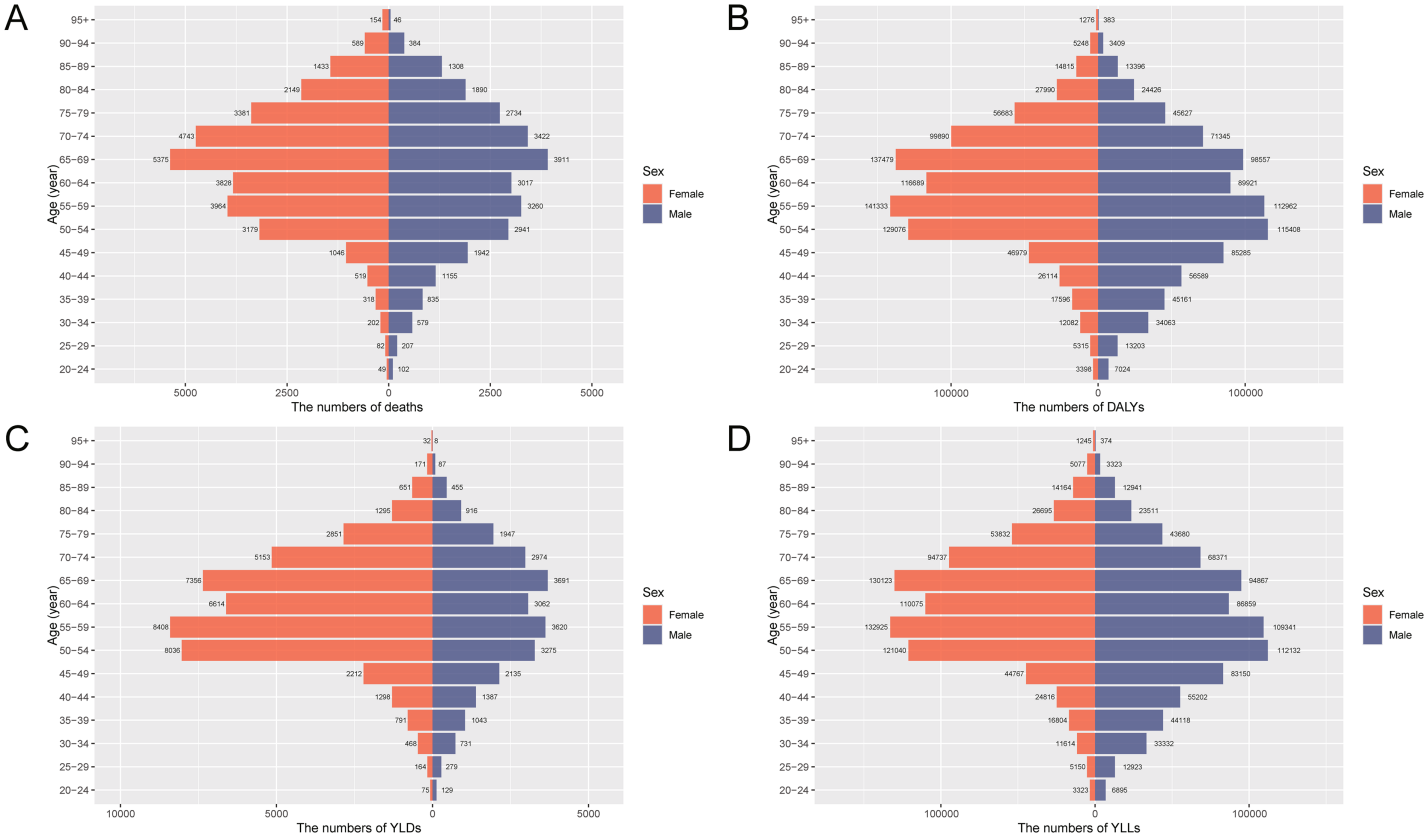
Age- and sex-specific numbers of deaths, DALYs, YLDs, and YLLs for total cancer attributable to high BMI in China in 2021. **(A)** Number of deaths attributable to high BMI by age group and sex. **(B)** Number of DALYs by age group and sex. **(C)** Number of YLDs by age group and sex. **(D)** Number of YLLs by age group and sex. DALYs, disability-adjusted life years; YLDs, years lived with disability; YLLs, years of life lost; BMI, body mass index.

**Figure 2:**
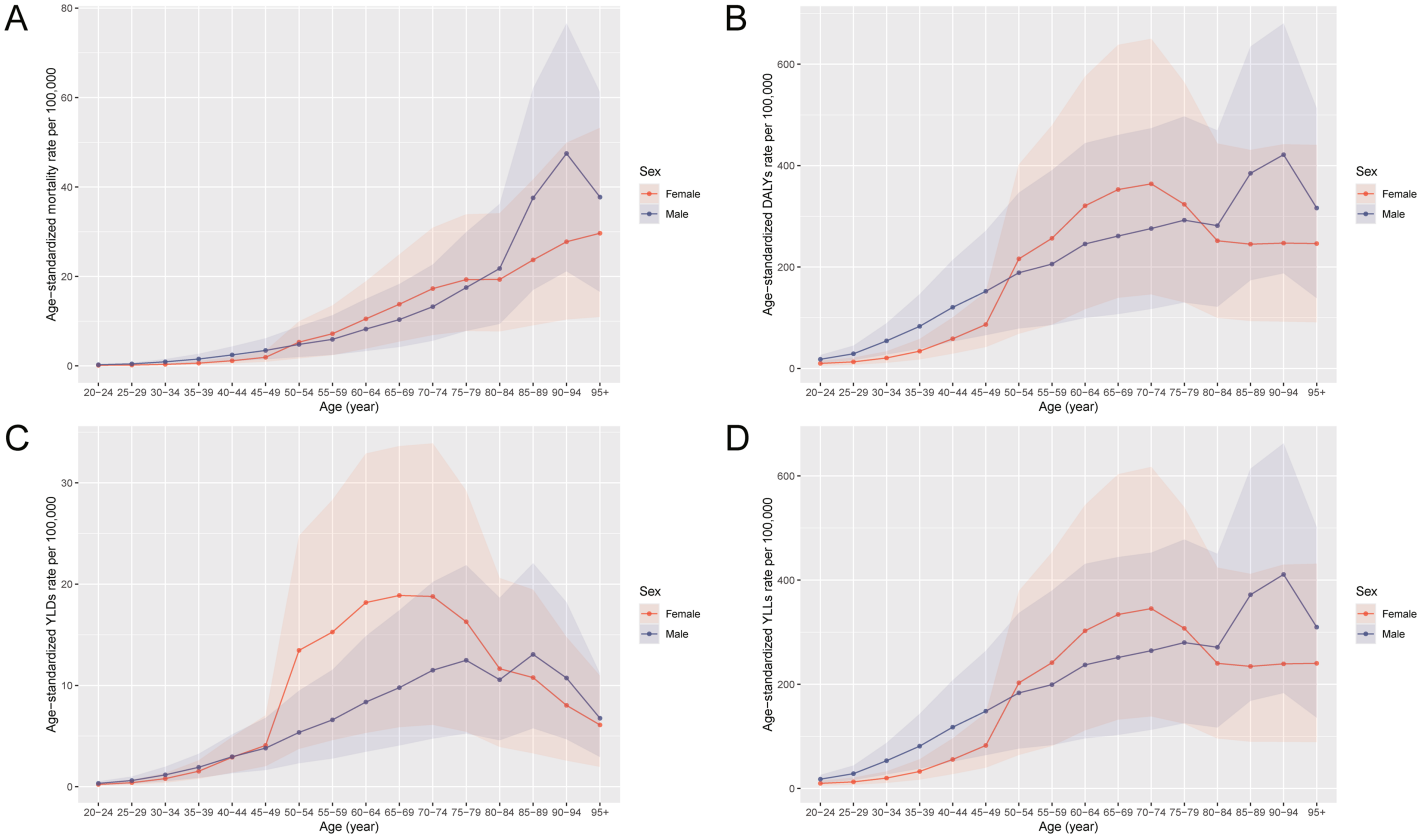
Age-specific and sex-specific rates of deaths, DALYs, YLDs, and YLLs for total cancer attributable to high BMI in China in 2021. **(A)** Age-standardized mortality rate per 100,000 population by age group and sex. **(B)** Age-standardized DALY rate per 100,000 population by age group and sex. **(C)** Age-standardized YLD rate per 100,000 population by age group and sex. **(D)** Age-standardized YLL rate per 100,000 population by age group and sex. DALYs, disability-adjusted life years; YLDs, years lived with disability; YLLs, years of life lost; BMI, body mass index.

The burden of total cancer attributable to high BMI in China in 2021 showed distinct patterns by age and sex (Figures 1, 2). In Figure 1, the number of deaths, DALYs, YLDs, and YLLs was lower for females than for males in individuals under 50 years of age. However, this trend reversed in the older population, with females showing higher absolute numbers than males in those aged 50 and above. For both sexes, the burden peaked between the ages of 50 and 69, indicating this age range as the most heavily affected group. Similarly, Figure 2 reveals that age-standardized rates of deaths, DALYs, and YLLs were higher in females than in males between the ages of 50 and 79, while YLDs showed higher rates in females between 50 and 84 years. In contrast, males had higher age-standardized rates across all indicators in the population under 50 years old and in those aged 80 years and above. These findings suggest that the cancer burden attributable to high BMI differs not only in magnitude but also in distribution between sexes across the lifespan, with women bearing a greater burden in the postmenopausal age range and men experiencing higher burden earlier and in older age.

### Temporal trends and age-specific changes in cancer burden attributable to high BMI in China from 1990 to 2021

**Figure 3:**
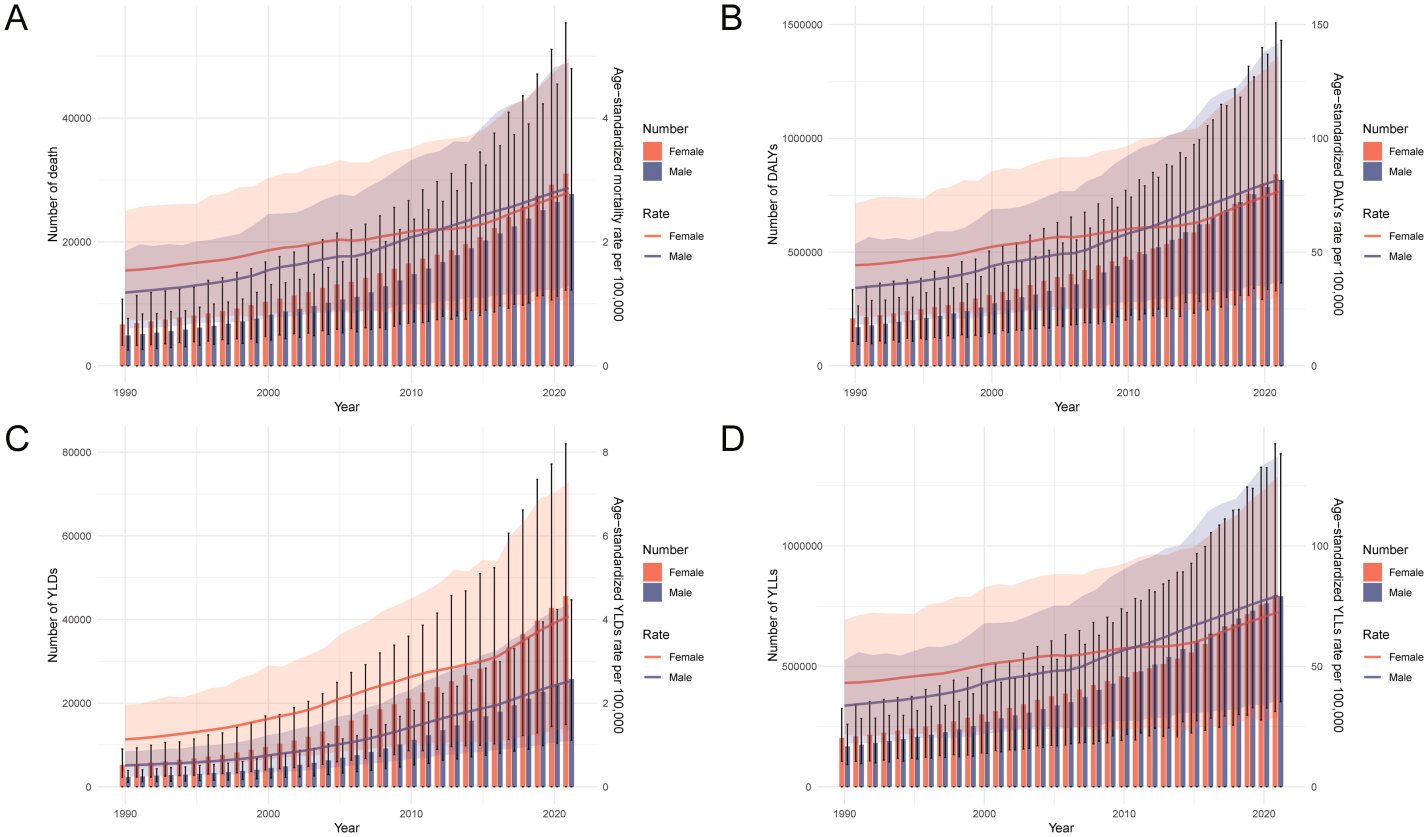
Temporal trends in the number and age-standardized rates of deaths, DALYs, YLDs, and YLLs for total cancer attributable to high BMI in China from 1990 to 2021. **(A)** Number and age-standardized mortality rate per 100,000 population by sex over time. **(B)** Number and age-standardized DALY rate per 100,000 population by sex over time. **(C)** Number and age-standardized YLL rate per 100,000 population by sex over time. **(D)** Number and age-standardized YLD rate per 100,000 population by sex over time. DALYs, disability-adjusted life years; YLDs, years lived with disability; YLLs, years of life lost; BMI, body mass index.

From 1990 to 2021, the burden of total cancer attributable to high BMI in China increased significantly in both absolute numbers and age-standardized rates across all major indicators, including deaths, DALYs, YLLs, and YLDs (Figure 3). The total number of cases rose steadily in both sexes, with a more pronounced increase observed in females. Notably, age-standardized rates of deaths, DALYs, and YLLs were higher in females than in males before 2012, but this pattern reversed thereafter, with males showing higher rates in the subsequent years. In contrast, the age-standardized YLD rate remained consistently higher in females throughout the entire period. These sex-specific shifts suggest changing epidemiological dynamics in the cancer burden related to high BMI. Supplementary Figure S1 further illustrates that between 1990 and 2021, both the number and crude rates of all indicators rose substantially across nearly all age groups, particularly among those aged 50–74 years. The increases were more pronounced in 2021, reflecting not only population aging but also an escalating impact of high BMI on cancer outcomes over time.

### Site-specific burden of cancer attributable to high BMI in 2021 and long-term trends

**Figure 4:**
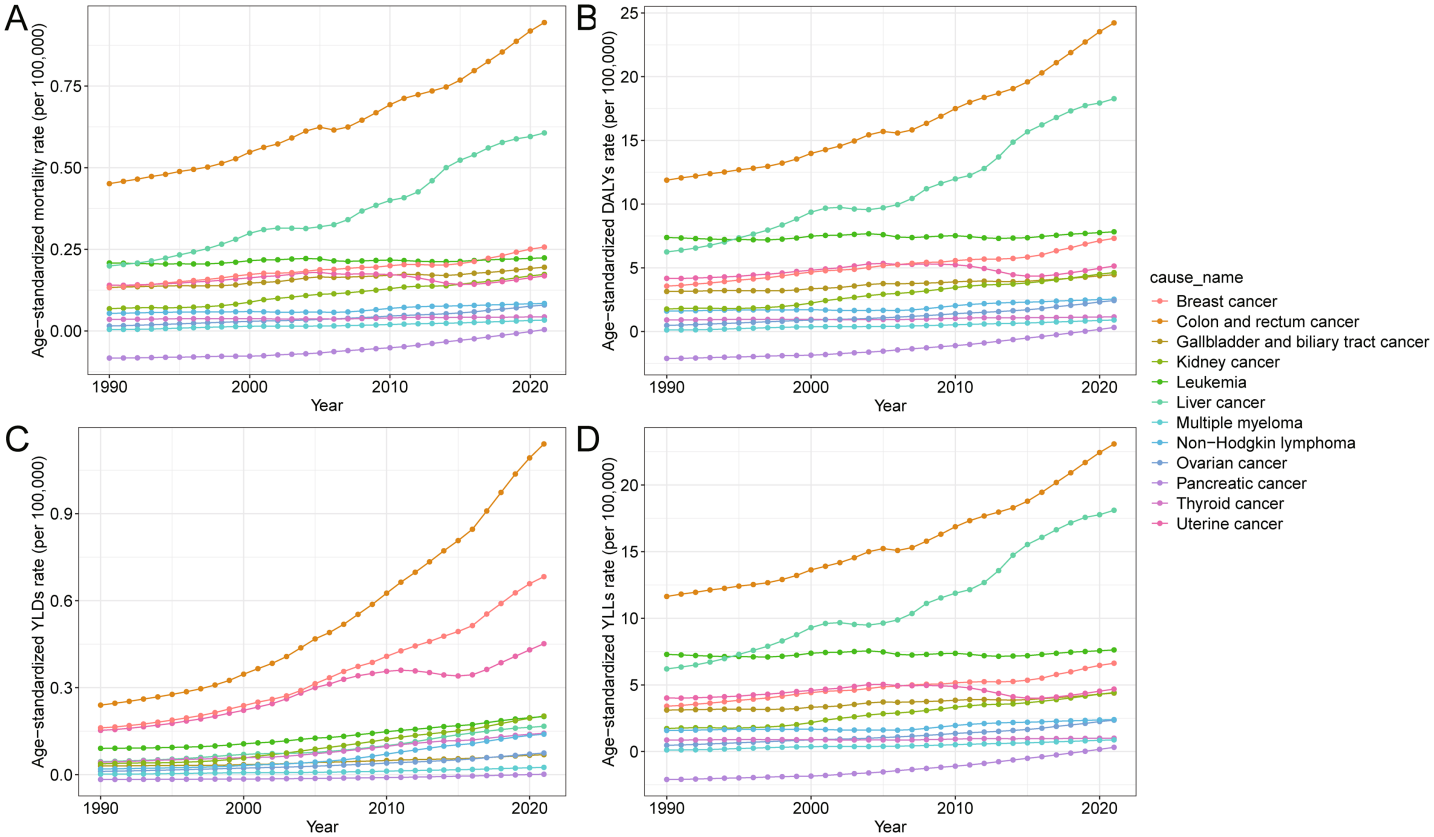
Temporal trends in age-standardized rates of mortality, DALYs, YLLs, and YLDs for site-specific cancers attributable to high BMI in China from 1990 to 2021. **(A)** Age-standardized mortality rate per 100,000 population over time for different cancer types. **(B)** Age-standardized DALY rate per 100,000 population over time for different cancer types. **(C)** Age-standardized YLL rate per 100,000 population over time for different cancer types. **(D)** Age-standardized YLD rate per 100,000 population over time for different cancer types. DALYs, disability-adjusted life years; YLDs, years lived with disability; YLLs, years of life lost; BMI, body mass index.

In 2021, among all site-specific cancers attributable to high BMI in China, colorectal cancer contributed the largest number of deaths (19.4 thousand) and DALYs (507.3 thousand), with substantial increases in age-standardized mortality (109.4%) and DALY rates (103.9%) from 1990 to 2021 (Table 2). Liver cancer followed, with 12.7 thousand deaths and 379.3 thousand DALYs, accompanied by the highest rise in age-standardized mortality (204.6%) and DALY rates (192.6%) among major cancers. Kidney cancer also exhibited marked increases, particularly in age-standardized DALYs (162.2%) and YLDs (438.9%), suggesting a growing burden associated with prolonged survival and disability (Supplementary Table S1). In contrast, pancreatic cancer was the only type showing declines in both mortality and DALYs, with negative growth in age-standardized rates. Female-specific cancers such as breast, uterine, and ovarian cancers showed moderate to substantial increases in both fatal (YLLs) and non-fatal (YLDs) burdens, particularly in breast cancer, where age-standardized DALY and YLD rates rose by over 100 and 322.8%, respectively. Figure 4 further illustrates the variation in age-standardized rates across different cancer types, highlighting that colorectal, liver, kidney, and breast cancers have become the dominant contributors to the total cancer burden attributable to high BMI. Supplementary Figure S2 further highlights marked sex differences in cancer burden. Males accounted for the majority of deaths and YLLs from cancers such as liver, colorectal, and kidney, while females bore nearly all the burden from breast, uterine, and ovarian cancers. Additionally, females had a higher proportion of YLDs for thyroid cancer and gallbladder and biliary tract cancer, indicating a greater non-fatal burden.

**Table 2:** Mortality and DALYs for total cancer attributable to high BMI in China, 2021, with trends in ASRs per 100,000 population, 1990–2021.

	Deaths			DALYs		
Cancer type	No, in thousands	Age-standardized rate per 100,000	Percentage change from 1990 to 2021	No, in thousands	Age-standardized rate per 100,000	Percentage change from 1990 to 2021
Total cancers	58.7 (24.6, 99.9)	2.8 (1.2, 4.8)	107 (53.8, 162.3)	1658.7 (693.2, 2831.3)	79.2 (33.8, 134.1)	102.3 (48.8, 159.7)
Colon and rectum cancer	19.4 (8.1, 32.5)	0.9 (0.4, 1.6)	109.4 (59.8, 196.7)	507.3 (209.3, 853.8)	24.2 (10, 40.7)	103.9 (54.8, 187.5)
Liver cancer	12.7 (5.1, 22.7)	0.6 (0.2, 1.1)	204.6 (113.7, 306.9)	379.3 (148.7, 695.9)	18.3 (7.2, 33.5)	192.6 (103.7, 300.7)
Gallbladder and biliary tract cancer	4.1 (2.3, 6)	0.2 (0.1, 0.3)	46.2 (7.5, 92)	95.6 (53.9, 142.8)	4.5 (2.5, 6.7)	41.8 (2.4, 87.7)
Pancreatic cancer	0.1 (−1.4, 2.8)	0 (−0.1, 0.1)	−104.9 (−412.9, −6.2)	7.7 (−32.8, 76.5)	0.3 (−1.6, 3.5)	−115 (−437.5, −13.5)
Kidney cancer	3.6 (1.3, 6.3)	0.2 (0.1, 0.3)	154.8 (96.3, 224.6)	96.7 (36, 170.3)	4.6 (1.7, 8.2)	162.2 (98.3, 238.4)
Thyroid cancer	0.9 (0.6, 1.2)	0 (0, 0.1)	21.4 (−4.5, 56.1)	23.7 (16.1, 32.5)	1.1 (0.8, 1.6)	26.2 (−0.6, 65.1)
Non-Hodgkin lymphoma	1.7 (0.6, 3.1)	0.1 (0, 0.1)	55.9 (15.1, 100.2)	51.9 (16.6, 91.8)	2.5 (0.8, 4.5)	58.3 (16.4, 105.6)
Multiple myeloma	0.7 (−0.2, 1.8)	0 (0, 0.1)	639.4 (179.5, 2089.3)	19.2 (−6.4, 49.1)	0.9 (−0.3, 2.3)	641 (173.9, 2025.6)
Leukemia	4.4 (2.9, 6.1)	0.2 (0.1, 0.3)	7.5 (−15.7, 37)	144 (96.9, 198.8)	7.8 (5.3, 10.8)	6 (−17.5, 35.7)
Breast cancer	5.7 (−0.2, 12.4)	0.3 (0, 0.6)	93.8 (37.6, 164.1)	169.4 (−5.5, 363.3)	7.3 (−0.2, 15.7)	105 (42.6, 184.2)
Uterine cancer	3.6 (2.2, 5.6)	0.2 (0.1, 0.3)	20.4 (−15, 71.1)	111 (66, 173.6)	5.1 (3.1, 8)	23.2 (−13.5, 77.2)
Ovarian cancer	1.7 (0.3, 3.6)	0.1 (0, 0.2)	422.8 (−1831, 2388.6)	53 (10.5, 108.3)	2.4 (0.5, 5)	409.9 (−1793.1, 2,834)

Values in parentheses indicate 95% UIs, estimated using Monte Carlo simulations. Extreme percentage changes may occur when baseline ASRs in 1990 are close to zero. DALYs, disability-adjusted life years; BMI, body mass index; ASRs, Age-standardized rates; UI, uncertainty interval.

### Joinpoint and APC analysis of trends in cancer burden attributable to high BMI in China from 1990 to 2021

**Figure 5:**
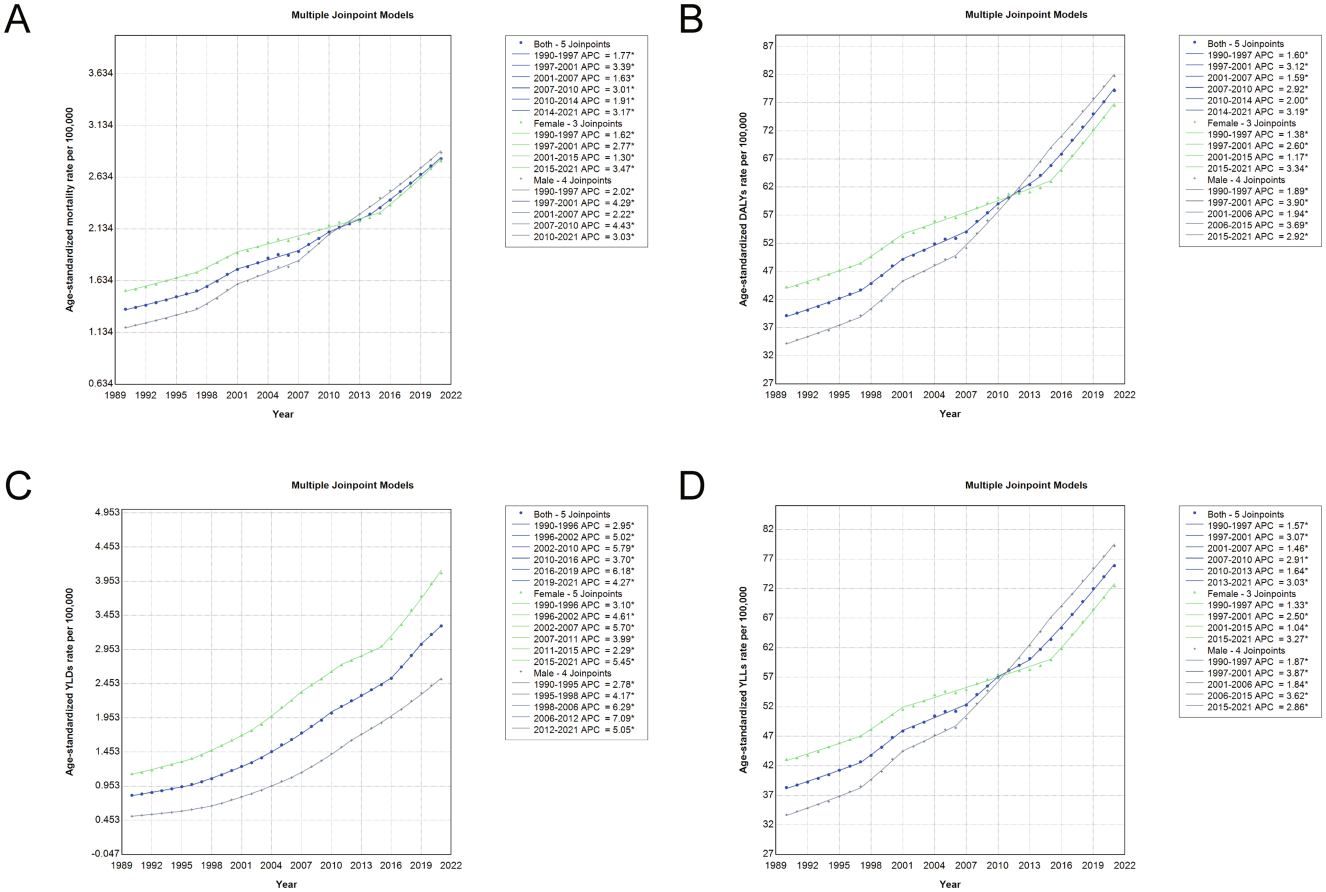
Joinpoint regression analysis of age-standardized rates for total cancer attributable to high BMI in China, 1990–2021. **(A)** Age-standardized mortality rate trends with APC and joinpoints. **(B)** Age-standardized DALY rate trends with APC and joinpoints. **(C)** Age-standardized YLD rate trends with APC and joinpoints. **(D)** Age-standardized YLL rate trends with APC and joinpoints. Each panel presents joinpoint regression trends for both sexes combined (blue line), females (green line), and males (gray line). Solid lines indicate fitted trends, and joinpoints represent statistically significant changes in trend direction over time. APC, annual percentage change; DALY, disability-adjusted life year; YLD, years lived with disability; YLL, years of life lost; BMI, body mass index.

Joinpoint regression analysis revealed distinct temporal patterns in the age-standardized burden of total cancer attributable to high BMI in China from 1990 to 2021 (Figure 5; Supplementary Table S2). Overall, the AAPC for age-standardized mortality, DALYs, YLDs, and YLLs rates showed significant upward trends in both sexes, with AAPCs of 2.40, 2.33, 4.62, and 2.25%, respectively. In the early years (1990–1997), mortality and DALYs rates grew steadily, followed by a sharper increase between 1997 and 2001. After 2014, another phase of accelerated growth was observed, particularly in YLDs, which surged between 2016 and 2019 (APC: 6.17%). Sex-specific analyses indicated that males experienced consistently higher AAPCs across most indicators compared to females, especially in the earlier periods. For example, the AAPC for age-standardized DALYs was 2.88% in males versus 1.82% in females, and the YLD rate rose by 5.30% annually in males compared to 4.27% in females. The age-standardized YLL rate also increased more rapidly in males. These trends demonstrate a sustained and accelerating rise in the cancer burden attributable to high BMI over the past three decades, with notable differences by sex and by period, highlighting the growing public health challenge posed by obesity-related cancers in China.

### Age-period-cohort effects on DALY and mortality rates of high BMI-related cancer in China

**Figure 6:**
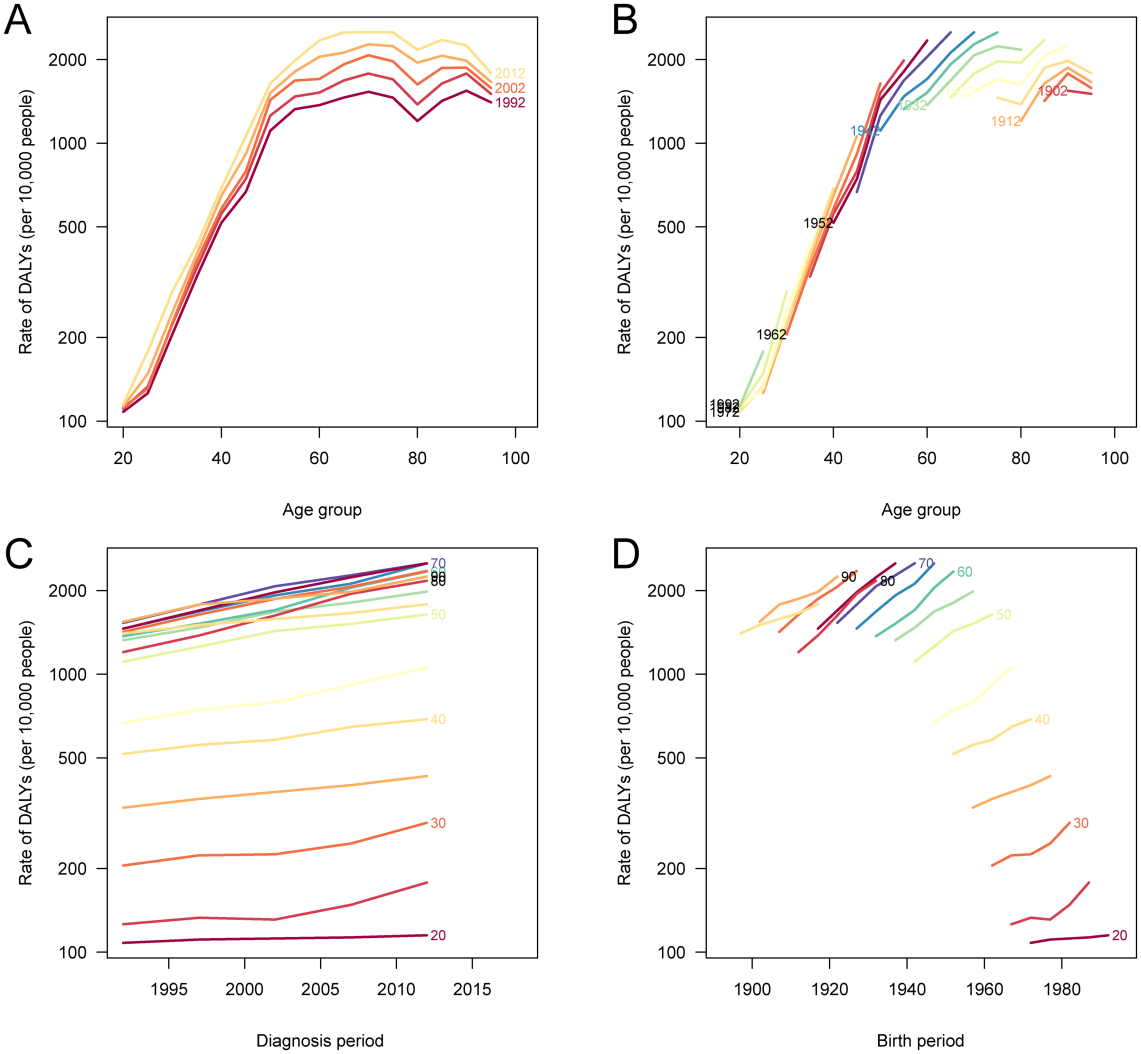
Age-period-cohort analysis of DALY rates for total cancer attributable to high BMI in China, 1990–2021. **(A)** The age-specific DALY rates according to calendar periods; each line connects the DALY rates for a given 5-year period. **(B)** The age-specific DALY rates according to birth cohorts; each line connects the DALY rates for a given 5-year birth cohort. **(C)** The period-specific DALY rates according to age groups; each line connects the DALY rates for a given 5-year age group. **(D)** The cohort-specific DALY rates according to age groups; each line connects the DALY rates for a given 5-year age group. DALY, disability-adjusted life year; BMI, body mass index.

The age-period-cohort analyses revealed important temporal patterns in DALY and mortality rates for total cancer attributable to high BMI in China from 1990 to 2021 (Figure 6; Supplementary Figure S3). Both DALY and mortality rates increased with age, peaking at 85–90 years for DALYs and 65–74 years for mortality, and then declined slightly in the oldest age groups. Period effects showed a consistent upward trend, with more recent calendar years associated with higher burden, suggesting the cumulative impact of rising BMI levels over time. Notably, the cohort-specific trends indicated that more recent birth cohorts experienced higher DALY and mortality rates compared to earlier cohorts, reflecting a shift in burden toward younger generations born after the 1960s. The local drift analyses confirmed that the annual percentage increases were most pronounced in the 55–74 age group, but the elevated burden among recent cohorts underscores the growing influence of high BMI on cancer risk across successive generations. These findings suggest that both aging and generational exposure to obesogenic environments are contributing to the escalating burden of BMI-related cancer in China.

### Drivers of change in cancer burden attributable to high BMI in China

**Figure 7:**
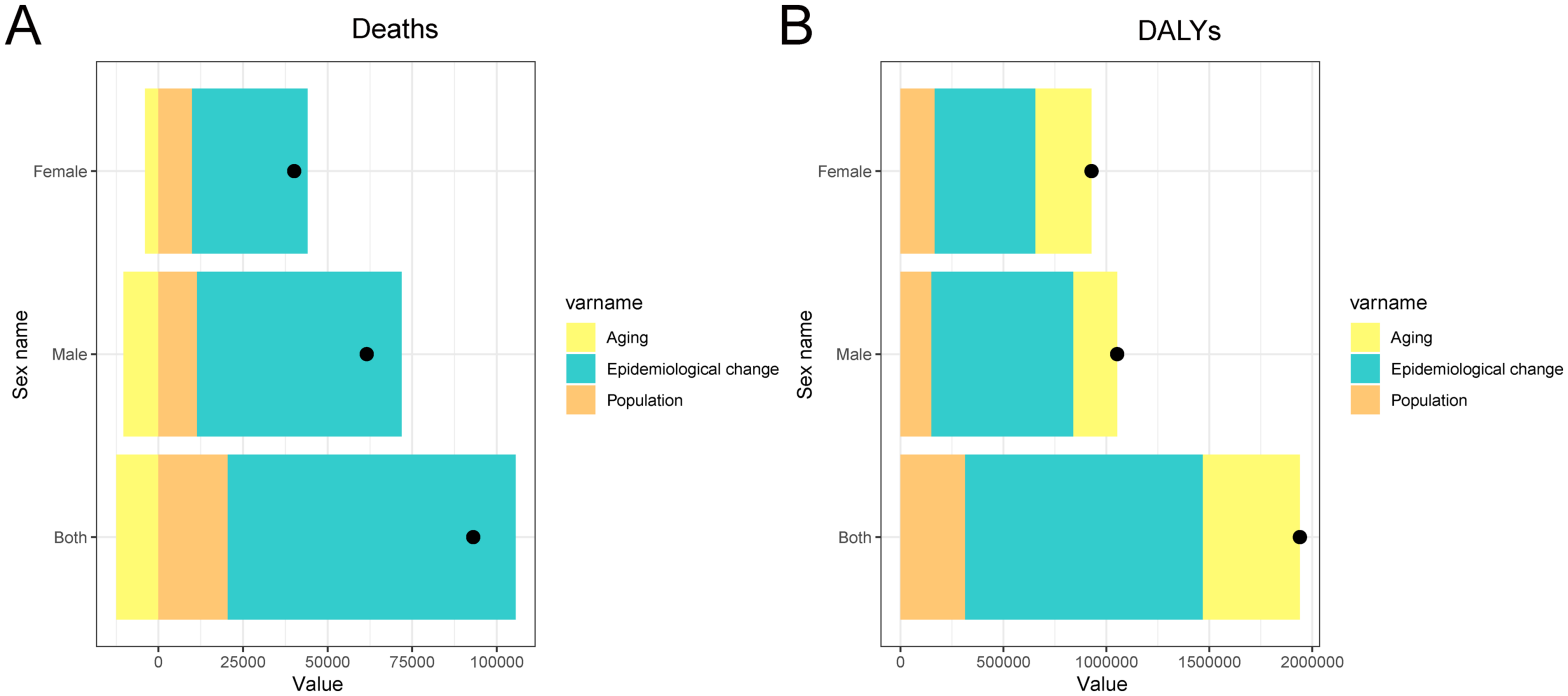
Decomposition analysis of changes in total cancer deaths and DALYs attributable to high BMI in China from 1990 to 2021. **(A)** Absolute contribution of population growth, population aging, and epidemiological changes to the increase in total DALYs. **(B)** Absolute contribution of population growth, population aging, and epidemiological changes to the increase in total deaths. DALY, disability-adjusted life year; BMI, body mass index.

The decomposition analysis revealed that the increase in cancer burden attributable to high BMI in China from 1990 to 2021 was driven by different combinations of epidemiological and demographic factors for deaths and DALYs, with notable sex differences (Figure 7). For deaths, the primary contributors were epidemiological change, reflecting shifts in exposure, disease risk, or treatment outcomes, and population growth. In contrast, population aging showed a negative contribution, suggesting that aging alone did not lead to more cancer deaths and may have offset some of the increase. For DALYs, all three components (epidemiological change, aging, and population growth) contributed to the rising burden, with epidemiological change again being the largest factor, followed by aging and population. Importantly, the effects of both aging and epidemiological change were more pronounced in males than females, indicating that men experienced a steeper rise in both fatal and non-fatal cancer outcomes associated with high BMI. These findings highlight the urgent need to address worsening risk exposures and reinforce prevention efforts, particularly among high-risk male populations.

## Discussion

This study presents a comprehensive overview of the burden and temporal trends of total cancer attributable to high BMI in China from 1990 to 2021, revealing a substantial and sustained increase over the past three decades. In 2021 alone, high BMI was estimated to be responsible for 58,745 cancer-related deaths and approximately 1.66 million DALYs, highlighting a significant public health concern. The burden was observed to be higher among males for mortality and YLLs, while females exhibited greater YLDs, indicating sex-specific differences in disease outcomes. Age-specific analyses showed that individuals aged 50–69 years bore the highest burden, and age-standardized rates rose more steeply in recent decades, particularly among men. Site-specific assessments identified colorectal, liver, kidney, and breast cancers as the dominant contributors to the total burden, with colorectal and liver cancers demonstrating the most rapid growth in age-standardized rates. Joinpoint regression revealed significant upward trends in all major indicators, and age-period-cohort analysis indicated rising DALY and mortality risks in more recent birth cohorts and calendar periods. Decomposition analysis showed that increases in deaths were largely driven by epidemiological changes and population growth, while DALY increases were most influenced by changes in age-specific rates, followed by aging and population expansion.

Collectively, these findings underscore the growing influence of high BMI on cancer burden in China and reveal complex temporal and demographic patterns that warrant targeted prevention and control strategies. Compared to Western countries, China has experienced a more rapid rise in BMI-related cancer burden in recent decades. While the global average annual increase in age-standardized mortality and DALY rates due to high BMI was modest (0.35 and 0.42%, respectively), China exhibited notably steeper increases, particularly after 2014, reflecting an accelerated accumulation of risk and disease onset. In contrast, high-SDI regions such as Western Europe and North America have shown plateauing or even declining trends in such burden, likely due to earlier implementation of effective obesity and cancer control programs (26). In China, rising high BMI-related cancers, including colorectal, liver, kidney, and uterine, generally mirror global patterns but are increasing more rapidly in this middle-income context. Furthermore, significant regional disparities in both obesity and cancer incidence have been documented within China, with higher obesity prevalence in northern and urbanized provinces and correspondingly greater burden of non-communicable diseases, including certain cancers (27). These patterns emphasize the need for region-specific strategies and underscore the urgency of early and sustained public health interventions. Drawing on lessons from countries where BMI-related cancer trends have stabilized, China’s national and subnational policies must address both the scale and heterogeneity of this growing challenge.

The biological mechanisms linking high BMI to increased cancer risk are multifactorial and well-documented. Excess adiposity induces a state of chronic low-grade inflammation, which promotes DNA damage, oxidative stress, and pro-tumorigenic signaling pathways (28, 29). Adipose tissue, particularly visceral fat, secretes a diverse array of bioactive molecules including cytokines, chemokines, and adipokines. These molecules play critical roles in modulating immune surveillance and remodeling the tumor microenvironment, thereby promoting cancer initiation and progression (29–31). Hyperinsulinemia and insulin resistance, common in individuals with obesity, further elevate cancer risk by increasing circulating insulin-like growth factor 1 (IGF-1), which has mitogenic and anti-apoptotic properties (32–34). Hormonal imbalances are also implicated: in postmenopausal women, elevated BMI is associated with increased peripheral conversion of androgens to estrogens in adipose tissue, raising the risk for estrogen receptor-positive breast and endometrial cancers (35, 36). Additionally, obesity alters bile acid metabolism and gut microbiota composition, which may contribute to hepatobiliary and colorectal carcinogenesis (37, 38). Notably, the relative importance of these mechanisms may vary by cancer type and sex; for example, estrogen-related hormonal pathways play a larger role in female-predominant cancers such as breast and uterine cancer, whereas insulin resistance and visceral adiposity may be more relevant to cancers more common in males, such as liver and colorectal cancer. Importantly, obesity may also influence cancer prognosis and treatment outcomes, as excess adiposity can impair chemotherapy efficacy, complicate surgical procedures, and increase the likelihood of comorbidities that affect treatment tolerance (39, 40). Collectively, these mechanisms highlight the systemic impact of high BMI on cancer biology and support the observed increase in BMI-related cancer burden in the Chinese population.

The observed differences in BMI-attributable cancer burden across sex, age, and cancer type reflect a complex interplay of biological, behavioral, and societal factors. The higher mortality and DALY rates in males may be explained by sex-based differences in fat distribution, with men more likely to accumulate visceral adipose tissue, which is more metabolically active and pro-inflammatory than subcutaneous fat (41–43). Additionally, lower cancer screening rates, delayed diagnosis, and higher prevalence of behavioral risk factors such as smoking and alcohol use in Chinese men may exacerbate the burden (44–46). Conversely, the higher YLD rates in females may result from earlier detection and better survival in cancers such as breast and thyroid, leading to longer periods of cancer-related disability. Age-wise, the burden peaked between ages 50–69, consistent with latency periods of obesity-related carcinogenesis and accumulation of metabolic damage. Decomposition analysis demonstrated that epidemiological shifts, manifested through deteriorating age-specific mortality rates, constituted the primary driver of increasing mortality and DALY burden. This finding underscores the critical role of rising BMI prevalence, alongside potential declines in physical activity levels and dietary quality, as key contributing factors. The age-period-cohort analysis also highlighted elevated burden among more recent birth cohorts, consistent with rising childhood and adolescent obesity in China since the 1990s (47). Compared with Western countries, where high BMI-attributable cancer burden has begun to plateau or decline due to stabilization of obesity rates and improved preventive measures (10), China appears to be at an earlier stage in the obesity-cancer trajectory, with rising trends paralleling rapid socioeconomic transition (48). These findings emphasize the urgency of early interventions and targeted public health responses.

The growing burden of total cancer attributable to high BMI in China presents urgent implications for both clinical practice and public health policy. Clinically, body weight should be systematically incorporated into cancer risk assessment, particularly for high-risk malignancies such as colorectal, liver, kidney, and postmenopausal breast cancers. Healthcare providers are encouraged to routinely assess BMI, deliver individualized lifestyle counseling, and integrate weight management into survivorship care planning, especially for middle-aged and elderly adults who face the highest burden. From a public health perspective, the rising age-standardized DALY rate, which captures the combined burden of early death and prolonged disability, serves as a compelling indicator of the urgent need for strengthened intervention efforts. This metric captures the cumulative impact of rising cancer incidence and survivorship burden and should guide resource prioritization. The findings support embedding obesity prevention within national cancer control plans through multisectoral strategies that promote healthy diets, physical activity, and health literacy. Early-life intervention is particularly essential, given the elevated burden in recent birth cohorts, likely driven by early exposure to obesogenic environments. Public health campaigns, school-based education, and structural changes such as active urban design should be prioritized. Furthermore, obesity should be addressed alongside other modifiable non-communicable disease (NCD) risk factors within an integrated national framework to enhance program efficiency and equity. As the obesity-linked cancer burden intensifies, especially in resource-limited areas, a coordinated response uniting clinical care, health systems, and policy reform is vital to reversing these trends and protecting future population health.

While this study provides important insights into the long-term burden and trends of total cancer attributable to high BMI in China, several limitations should be acknowledged. First, as the analysis was based on data from the GBD 2021, the estimates rely on complex modeling techniques that integrate data from multiple sources of varying quality, which may introduce uncertainty and potential bias (6). While state-of-the-art modeling frameworks like DisMod-MR and CODEm are employed to account for inconsistencies, limitations in primary data availability, especially from rural and underserved regions, may compromise the accuracy of burden estimates. Second, high BMI was treated as a single, continuous exposure without accounting for differences in fat distribution (e.g., visceral vs. subcutaneous adiposity), duration of obesity, or metabolic health status, which are increasingly recognized as important modifiers of cancer risk (49). Third, the comparative risk assessment framework employed by GBD includes only cancer types with strong epidemiologic evidence linking them to high BMI; emerging associations with additional malignancies may not yet be reflected, potentially leading to an underestimation of the total burden. Fourth, the use of IOTF standards for children and adolescents, though globally accepted, may not perfectly align with Chinese-specific growth references, potentially introducing classification bias in youth populations. Moreover, the ecological nature of the study prevents causal inferences and limits the ability to adjust for individual-level confounders such as physical activity, diet, genetics, or access to healthcare. Future research should incorporate longitudinal cohort data to explore causal mechanisms, better account for biological heterogeneity, and examine intervention effects. Additionally, region-specific analyses are needed to assess disparities in BMI-related cancer burden across China’s diverse socioeconomic and geographic contexts.

## Conclusion

This study reinforces the urgent need to address high BMI as a critical and modifiable risk factor within the broader framework of cancer prevention and control in China. As the country undergoes rapid demographic and lifestyle transitions, the burden of BMI-related cancer including colorectal, liver, kidney, and postmenopausal breast cancers is likely to continue rising unless comprehensive and sustained interventions are implemented. Effective responses will require a multidisciplinary approach that integrates clinical care, public health policy, and health education. Future research should aim to clarify the biological and behavioral pathways linking obesity and cancer, with a particular focus on sex- and age-specific vulnerabilities. Additionally, greater emphasis should be placed on evaluating the effectiveness of population-based obesity prevention strategies in reducing long-term cancer outcomes. Attention should also be given to colorectal, liver, kidney, and breast cancers as they represent the most significantly impacted cancer types. Regional and socioeconomic disparities also warrant further investigation to inform equitable health policies. Strengthening surveillance systems and leveraging prospective cohort data will be essential for guiding targeted, evidence-based interventions in the coming decades.

## Data Availability

Publicly available datasets were analyzed in this study. This data can be found at: http://ghdx.healthdata.org/gbd-results-tool.

## Footnotes

^1^ http://ghdx.healthdata.org/gbd-results-tool

## Supplementary material

Figure S1 Comparison of the number and crude rates of deaths, DALYs, YLDs, and YLLs for total cancer attributable to high BMI in China by age group in 1990 and 2021. **(A)** Number and crude mortality rate per 100,000 population by age group in 1990 and 2021. **(B)** Number and crude DALY rate per 100,000 population by age group in 1990 and 2021. **(C)** Number and crude YLL rate per 100,000 population by age group in 1990 and 2021. **(D)** Number and crude YLD rate per 100,000 population by age group in 1990 and 2021. DALYs, disability-adjusted life years; YLDs, years lived with disability; YLLs, years of life lost; BMI, body mass index.

Figure S2 Sex distribution of site-specific cancer burden attributable to high BMI in China in 2021. **(A)** Sex distribution by cause for deaths. **(B)** Sex distribution by cause for DALYs. **(C)** Sex distribution by cause for YLLs. **(D)** Sex distribution by cause for YLDs. DALYs, disability-adjusted life years; YLDs, years lived with disability; YLLs, years of life lost; BMI, body mass index.

Figure S3 Age-period-cohort analysis of mortality rates for total cancer attributable to high BMI in China, 1990-2021. **(A)** The age-specific mortality rates according to calendar periods; each line connects the mortality rates for a given 5-year period. **(B)** The age-specific mortality rates according to birth cohorts; each line connects the mortality rates for a given 5-year birth cohort. **(C)** The period-specific mortality rates according to age groups; each line connects the mortality rates for a given 5-year age group. **(D)** The cohort-specific mortality rates according to age groups; each line connects the mortality rates for a given 5-year age group. BMI, body mass index.

## References

[1] Ng M Gakidou E Lo J Abate YH Abbafati C Abbas N. Global, regional, and national prevalence of adult overweight and obesity, 1990–2021, with forecasts to 2050: a forecasting study for the global burden of disease study 2021. Lancet. (2025) 405:813–38. doi: 10.1016/S0140-6736(25)00355-1, PMID: 40049186 PMC11920007

[2] Islam ANMS Sultana H Nazmul Hassan Refat M Farhana Z Abdulbasah Kamil A Meshbahur Rahman M. The global burden of overweight-obesity and its association with economic status, benefiting from STEPs survey of WHO member states: A meta-analysis. Prev Med Rep. (2024) 46:102882. doi: 10.1016/j.pmedr.2024.102882, PMID: 39290257 PMC11406007

[3] Wang L Zhou B Zhao Z Yang L Zhang M Jiang Y. Body-mass index and obesity in urban and rural China: findings from consecutive nationally representative surveys during 2004-18. Lancet (London, England). (2021) 398:53–63. doi: 10.1016/S0140-6736(21)00798-4, PMID: 34217401 PMC7617101

[4] Huang L Wang Z Wang H Zhao L Jiang H Zhang B. Nutrition transition and related health challenges over decades in China. Eur J Clin Nutr. (2021) 75:247–52. doi: 10.1038/s41430-020-0674-8 32620907

[5] Howard AG Attard SM Herring AH Wang H Du S Gordon-Larsen P. Socioeconomic gradients in the westernization of diet in China over 20 years. SSM Popul Health. (2021) 16:100943. doi: 10.1016/j.ssmph.2021.100943, PMID: 34703875 PMC8526760

[6] GBD 2021 Risk Factors Collaborators. Global burden and strength of evidence for 88 risk factors in 204 countries and 811 subnational locations, 1990-2021: a systematic analysis for the global burden of disease study 2021. Lancet (London, England). (2024) 403:2162–203. doi: 10.1016/S0140-6736(24)00933-4 38762324 PMC11120204

[7] Zhou X-D Chen Q-F Yang W Zuluaga M Targher G Byrne CD. Burden of disease attributable to high body mass index: an analysis of data from the global burden of disease study 2021. eClinicalMedicine. (2024) 76:102848. doi: 10.1016/j.eclinm.2024.102848, PMID: 39386160 PMC11462227

[8] Zhan Z Chen X Xu S Li Q Yu J Guo Z. Impact of high body mass index on gallbladder and biliary tract cancer burden in China: a comprehensive analysis of trends from 1990 to 2021. World J Surg Oncol. (2024) 22:296. doi: 10.1186/s12957-024-03582-4, PMID: 39529095 PMC11556143

[9] Lauby-Secretan B Scoccianti C Loomis D Grosse Y Bianchini F Straif K. Body fatness and Cancer — viewpoint of the IARC working group. N Engl J Med. (2016) 375:794–8. doi: 10.1056/NEJMsr1606602, PMID: 27557308 PMC6754861

[10] Tan DJH Ng CH Muthiah M Yong JN Chee D Teng M. Rising global burden of cancer attributable to high BMI from 2010 to 2019. Metab Clin Exp. (2024) 152:155744. doi: 10.1016/j.metabol.2023.155744, PMID: 38029839 PMC11321712

[11] Larsson SC Spyrou N Mantzoros CS. Body fatness associations with cancer: evidence from recent epidemiological studies and future directions. Metab Clin Exp. (2022) 137:155326. doi: 10.1016/j.metabol.2022.155326, PMID: 36191637

[12] Fosam A Perry RJ. Current mechanisms in obesity and tumor progression. Curr Opin Clin Nutr Metab Care. (2020) 23:395–403. doi: 10.1097/MCO.0000000000000690, PMID: 32868685 PMC10059279

[13] Ahmed M Mulugeta A Lee SH Mäkinen V-P Boyle T Hyppönen E. Adiposity and cancer: a Mendelian randomization analysis in the UK biobank. Int J Obes. (2021) 45:2657–65. doi: 10.1038/s41366-021-00942-y, PMID: 34453097

[14] Kim MS Song M Kim S Kim B Kang W Kim JY. Causal effect of adiposity on the risk of 19 gastrointestinal diseases: a Mendelian randomization study. Obesity (Silver Spring, Md). (2023) 31:1436–44. doi: 10.1002/oby.23722, PMID: 37014069 PMC10192008

[15] Xing A Tong HHY Liu S Zhai X Yu L Li K. The causal association between obesity and gastric cancer and shared molecular signatures: a large-scale Mendelian randomization and multi-omics analysis. Front Oncol. (2023) 13:1091958. doi: 10.3389/fonc.2023.1091958, PMID: 37954072 PMC10639150

[16] Crispo A Augustin LSA Luongo A Calderaio C Breda J Coluccia S. Central obesity, body mass index, metabolic syndrome and mortality in Mediterranean breast cancer patients. Sci Rep. (2023) 13:21208. doi: 10.1038/s41598-023-45439-y, PMID: 38040773 PMC10692221

[17] Tu H McQuade JL Davies MA Huang M Xie K Ye Y. Body mass index and survival after cancer diagnosis: A pan-cancer cohort study of 114 430 patients with cancer. Innovation. (2022) 3:100344. doi: 10.1016/j.xinn.2022.100344, PMID: 36353671 PMC9638833

[18] Arnold M Leitzmann M Freisling H Bray F Romieu I Renehan A. Obesity and cancer: An update of the global impact. Cancer Epidemiol. (2016) 41:8–15. doi: 10.1016/j.canep.2016.01.003, PMID: 26775081

[19] Murray CJL. Findings from the global burden of disease study 2021. Lancet. (2024) 403:2259–62. doi: 10.1016/S0140-6736(24)00769-4 38762327

[20] Zhan Z Chen B Zeng Y Huang R Yu J Guo Z. Long-term trends and projections of stomach cancer burden in China: insights from the GBD 2021 study. PLoS One. (2025) 20:e0320751. doi: 10.1371/journal.pone.0320751, PMID: 40198592 PMC11978042

[21] Kim HJ Fay MP Feuer EJ Midthune DN. Permutation tests for joinpoint regression with applications to cancer rates. Stat Med. (2000) 19:335–51. doi: 10.1002/(SICI)1097-0258(20000215)19:3<335::AID-SIM336>3.0.CO;2-Z, PMID: 10649300

[22] Zhan Z Chen X Zheng J Xu J Zhou S Guo Z. Burden of colon and rectum cancer attributable to processed meat consumption in China, 1990-2021. Front Nutr. (2025) 12:1488077. doi: 10.3389/fnut.2025.1488077, PMID: 40225336 PMC11985440

[23] Carstensen B. Age-period-cohort models for the Lexis diagram. Stat Med. (2007) 26:3018–45. doi: 10.1002/sim.2764, PMID: 17177166

[24] Rosenberg PS Miranda-Filho A. Advances in statistical methods for cancer surveillance research: an age-period-cohort perspective. Front Oncol. (2023) 13:1332429. doi: 10.3389/fonc.2023.1332429, PMID: 38406174 PMC10889111

[25] Zhan Z Chen B Lin W Chen X Huang R Yang C. Rising burden of Colon and Rectum Cancer in China: An analysis of trends, gender disparities, and projections to 2030. Ann Surg Oncol. (2025) 32:3361–71. doi: 10.1245/s10434-025-16905-w 39836276

[26] Figlioli G Piovani D Tsantes AG Pugliese N Nikolopoulos GK Hassan C. Burden of cancer attributable to high body mass index: A systematic analysis of the global burden of disease study 2021. Clin Nutr (Edinburgh, Scotland). (2025) 48:144–52. doi: 10.1016/j.clnu.2025.04.002 40215883

[27] Zhang L Chen J Zhang J Wu W Huang K Chen R. Regional disparities in obesity among a heterogeneous population of Chinese children and adolescents. JAMA Netw Open. (2021) 4:e2131040. doi: 10.1001/jamanetworkopen.2021.31040, PMID: 34698846 PMC8548942

[28] Kawai T Autieri MV Scalia R. Adipose tissue inflammation and metabolic dysfunction in obesity. Am J Physiol Cell Physiol. (2021) 320:C375–c391. doi: 10.1152/ajpcell.00379.2020, PMID: 33356944 PMC8294624

[29] Uti DE Atangwho IJ Omang WA Alum EU Obeten UN Udeozor PA. Cytokines as key players in obesity low grade inflammation and related complications. Obes Med. (2025) 54:100585. doi: 10.1016/j.obmed.2025.100585

[30] Martínez-Fernández L Fernández-Galilea M Felix-Soriano E Escoté X González-Muniesa P Moreno-Aliaga MJ: Chapter 4 - inflammation and oxidative stress in adipose tissue: nutritional regulation. In: Obesity. Edited by Moral AM del Aguilera García CM. Cambridge, Massachusetts, USA: Academic Press; (2018), 63–92.

[31] Himbert C Delphan M Scherer D Bowers LW Hursting S Ulrich CM. Signals from the adipose microenvironment and the obesity-Cancer link-A systematic review. Cancer Prev Res (Phila). (2017) 10:494–506. doi: 10.1158/1940-6207.CAPR-16-0322, PMID: 28864539 PMC5898450

[32] Zhang AMY Wellberg EA Kopp JL Johnson JD. Hyperinsulinemia in obesity, inflammation, and Cancer. Diabetes Metab J. (2021) 45:285–311. doi: 10.4093/dmj.2020.0250, PMID: 33775061 PMC8164941

[33] Gallagher EJ LeRoith D. Hyperinsulinaemia in cancer. Nat Rev Cancer. (2020) 20:629–44. doi: 10.1038/s41568-020-0295-5, PMID: 32908223

[34] Renehan AG Zwahlen M Egger M. Adiposity and cancer risk: new mechanistic insights from epidemiology. Nat Rev Cancer. (2015) 15:484–98. doi: 10.1038/nrc3967, PMID: 26205341

[35] Ding S Madu CO Lu Y. The impact of hormonal imbalances associated with obesity on the incidence of endometrial Cancer in postmenopausal women. J Cancer. (2020) 11:5456–65. doi: 10.7150/jca.47580, PMID: 32742493 PMC7391192

[36] Kuryłowicz A. Estrogens in adipose tissue physiology and obesity-related dysfunction. Biomedicines. (2023) 11:690. doi: 10.3390/biomedicines11030690, PMID: 36979669 PMC10045924

[37] Collins SL Stine JG Bisanz JE Okafor CD Patterson AD. Bile acids and the gut microbiota: metabolic interactions and impacts on disease. Nat Rev Microbiol. (2023) 21:236–47. doi: 10.1038/s41579-022-00805-x, PMID: 36253479 PMC12536349

[38] Zhao Q Wu J Ding Y Pang Y Jiang C. Gut microbiota, immunity, and bile acid metabolism: decoding metabolic disease interactions. Life. Metabolism. (2023) 2:load032. doi: 10.1093/lifemeta/load032, PMID: 39872860 PMC11749371

[39] Slawinski CGV Barriuso J Guo H Renehan AG. Obesity and Cancer treatment outcomes: interpreting the complex evidence. Clin Oncol (R Coll Radiol). (2020) 32:591–608. doi: 10.1016/j.clon.2020.05.004 32595101

[40] Lysaght J Conroy MJ. The multifactorial effect of obesity on the effectiveness and outcomes of cancer therapies. Nat Rev Endocrinol. (2024) 20:701–14. doi: 10.1038/s41574-024-01032-5, PMID: 39313571

[41] Małodobra-Mazur M Cierzniak A Pawełka D Kaliszewski K Rudnicki J Dobosz T. Metabolic differences between subcutaneous and visceral adipocytes differentiated with an excess of saturated and monounsaturated fatty acids. Genes. (2020) 11:1092. doi: 10.3390/genes11091092, PMID: 32962087 PMC7563871

[42] Nauli AM Matin S. Why Do men accumulate abdominal visceral fat? Front Physiol. (2019) 10:1486. doi: 10.3389/fphys.2019.01486, PMID: 31866877 PMC6906176

[43] Zhan Z Chen B Huang R Lin W Lan S Yao X. Long-term trends and future projections of liver cancer burden in China from 1990 to 2030. Sci Rep. (2025) 15:13120. doi: 10.1038/s41598-025-96615-1, PMID: 40240432 PMC12003824

[44] Liu K Ding Y Lu X Wang Z. Trends and socioeconomic factors in smoking and alcohol consumption among Chinese people: evidence from the 2008–2018 National Health Service Surveys in Jiangsu Province. Arch Public Health. (2021) 79:127. doi: 10.1186/s13690-021-00646-9, PMID: 34243791 PMC8268563

[45] Xia C Basu P Kramer BS Li H Qu C Yu XQ. Cancer screening in China: a steep road from evidence to implementation. Lancet Public Health. (2023) 8:e996–e1005. doi: 10.1016/S2468-2667(23)00186-X, PMID: 38000379 PMC10665203

[46] Zeng H Ran X An L Zheng R Zhang S Ji JS. Disparities in stage at diagnosis for five common cancers in China: a multicentre, hospital-based, observational study. Lancet Public Health. (2021) 6:e877–87. doi: 10.1016/S2468-2667(21)00157-2, PMID: 34838194

[47] Kerr JA Patton GC Cini KI Abate YH Abbas N Abd Al Magied AHA. Global, regional, and national prevalence of child and adolescent overweight and obesity, 1990–2021, with forecasts to 2050: a forecasting study for the global burden of disease study 2021. Lancet. (2025) 405:785–812. doi: 10.1016/S0140-6736(25)00397-6, PMID: 40049185 PMC11920006

[48] Hemmingsson E. The unparalleled rise of obesity in China: a call to action. Int J Obes. (2021) 45:921–2. doi: 10.1038/s41366-021-00774-w, PMID: 33608648

[49] Loh NY Wang W Noordam R Christodoulides C. Obesity, fat distribution and risk of cancer in women and men: A Mendelian randomisation study. Nutrients. (2022) 14:5259. doi: 10.3390/nu14245259, PMID: 36558416 PMC9784937

