# Supplementary figures and images for: Long-term trends in the burden of cancer attributable to high body mass index in China from 1990 to 2021
Source: Front Nutr. 2025 May 21;12:1606747. doi: 10.3389/fnut.2025.1606747 (PMC12133465; doi:10.3389/fnut.2025.1606747)

A

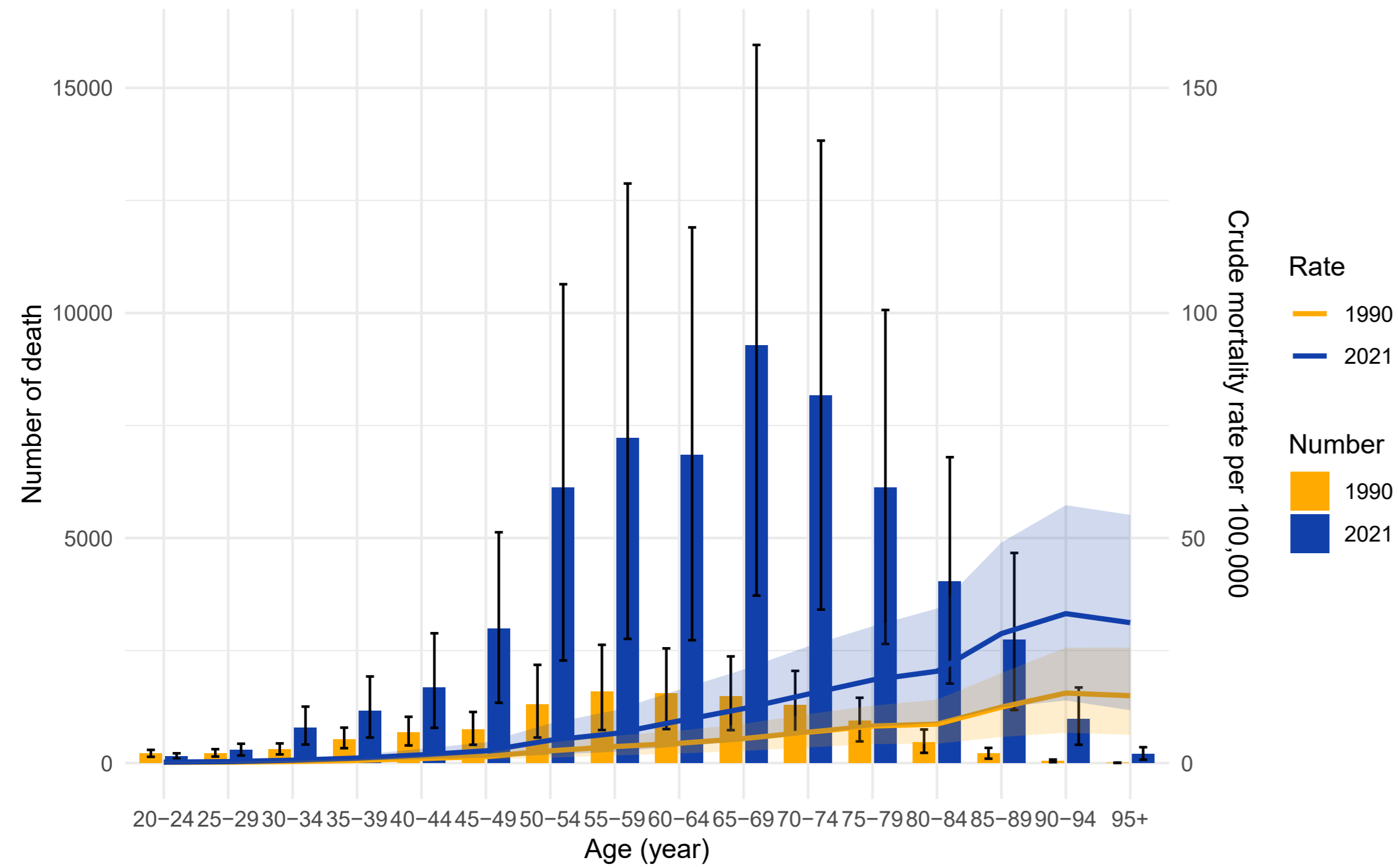

B

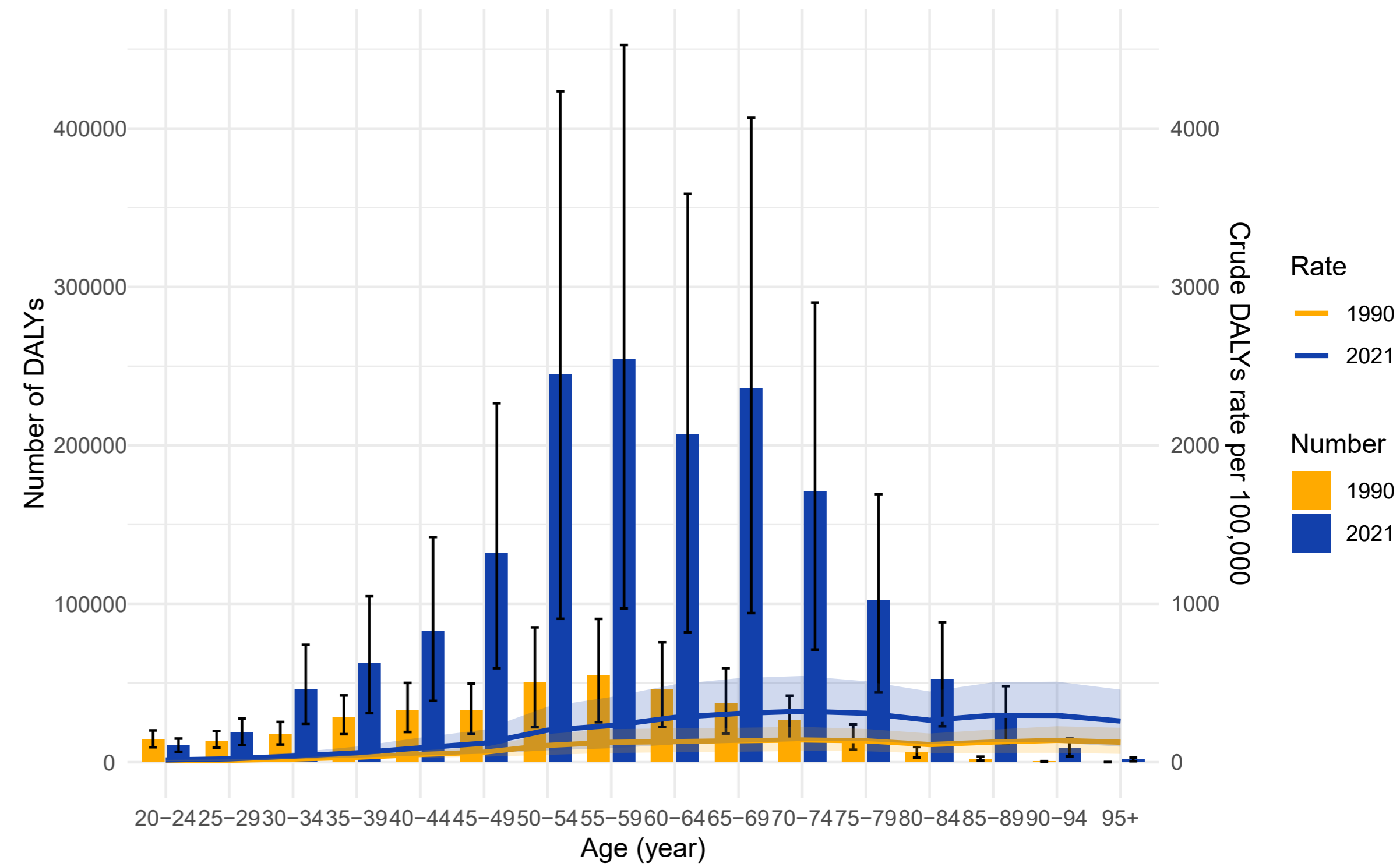

C

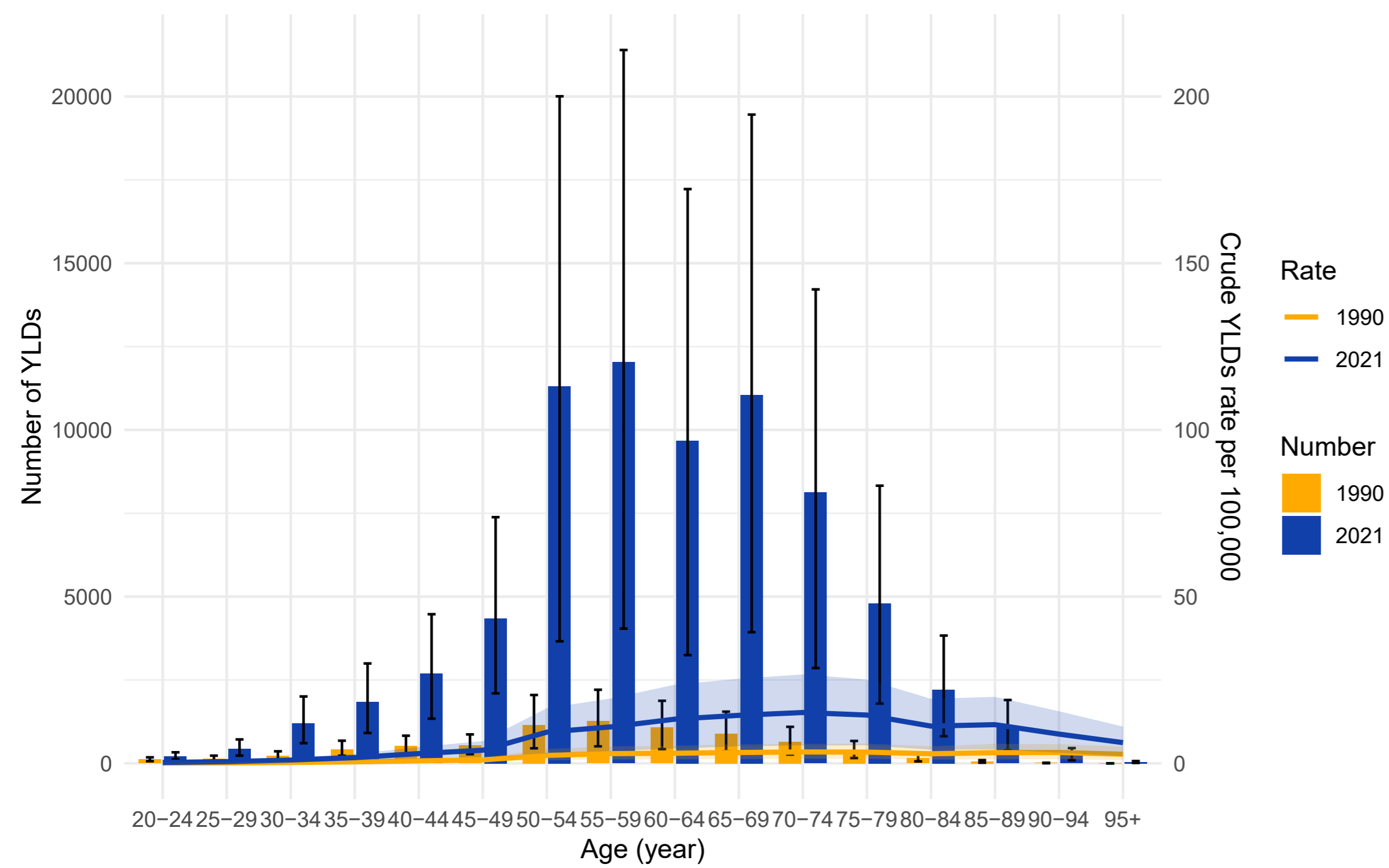

D

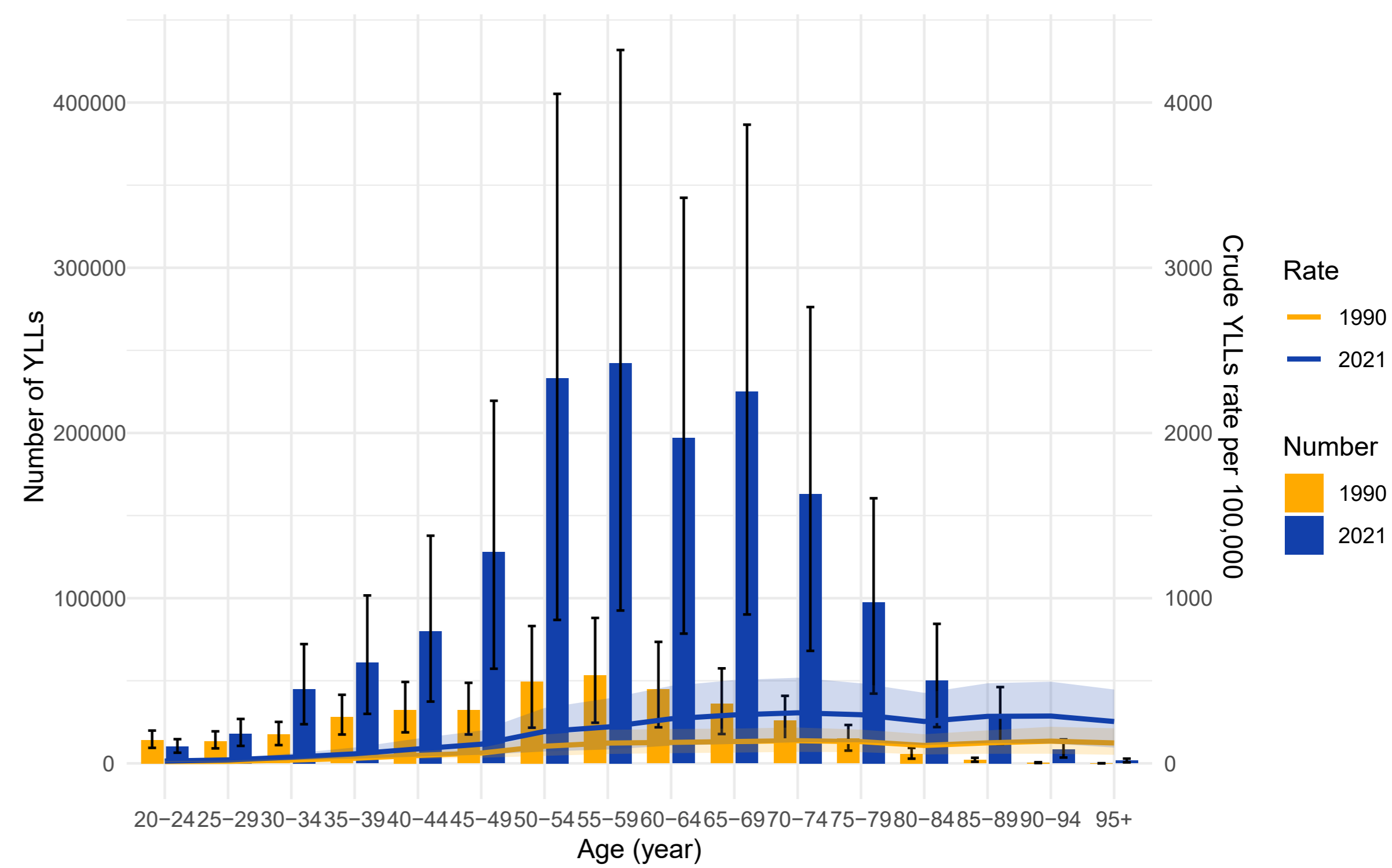

Supplement: Figure S1 — Comparison of the number and crude rates of deaths, DALYs, YLDs, and YLLs for total cancer attributable to high BMI in China by age group in 1990 and 2021. (A) Number and crude mortality rate per 100,000 population by age group in 1990 and 2021. (B) Number and crude DALY rate per 100,000 population by age group in 1990 and 2021. (C) Number and crude YLL rate per 100,000 population by age group in 1990 and 2021. (D) Number and crude YLD rate per 100,000 population by age group in 1990 and 2021. DALYs, disability-adjusted life years; YLDs, years lived with disability; YLLs, years of life lost; BMI, body mass index. [file Image_1.pdf]

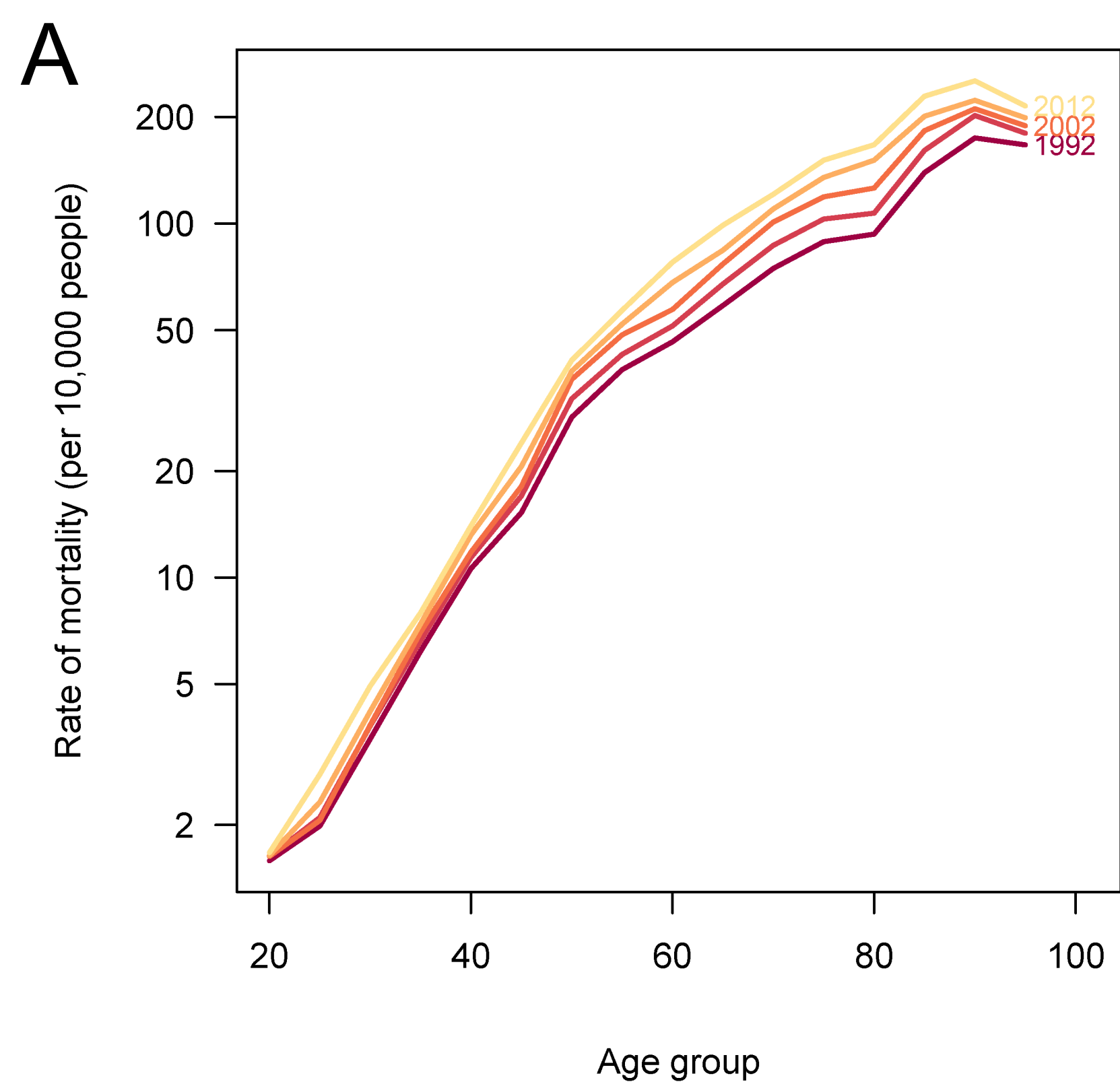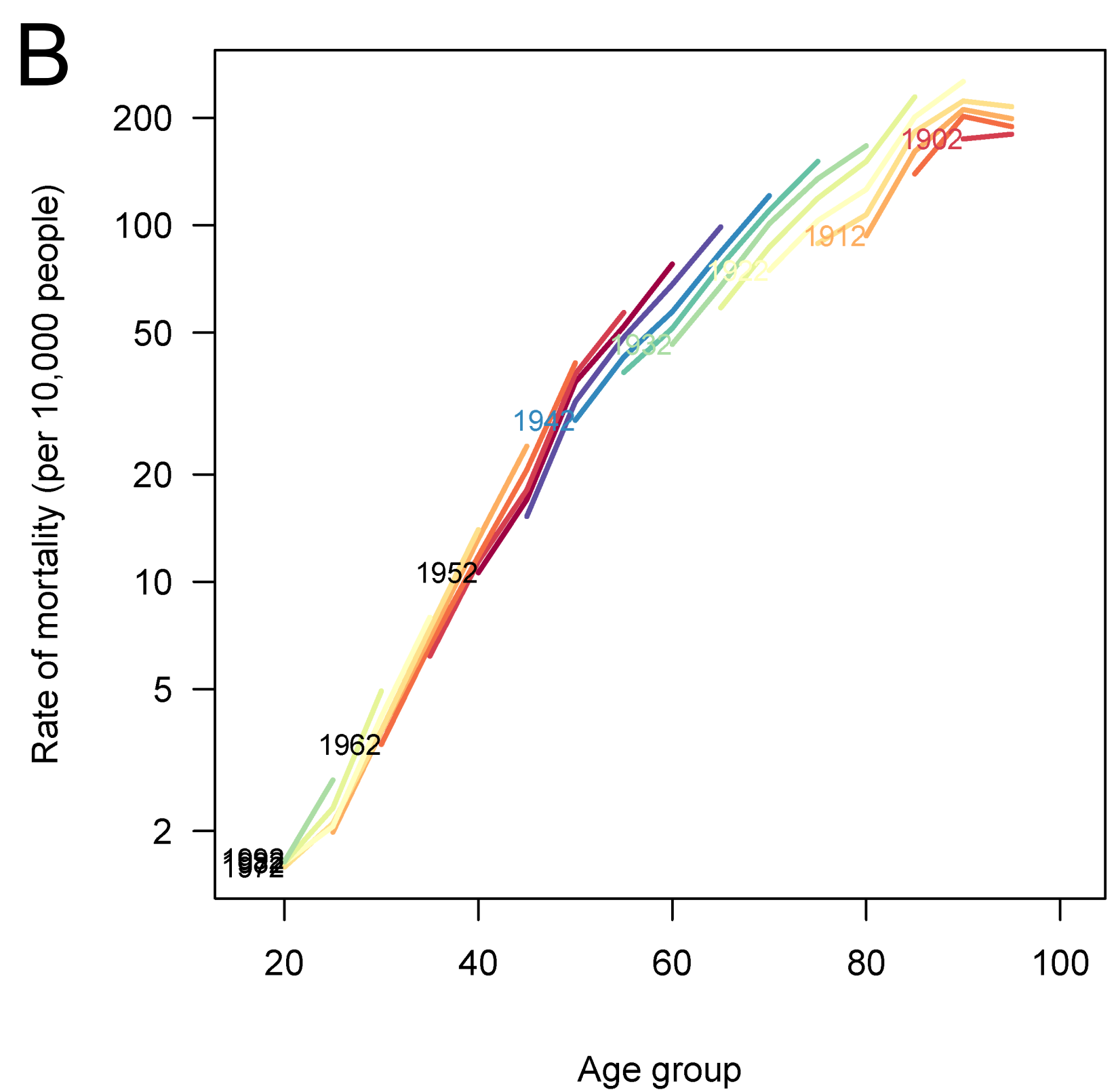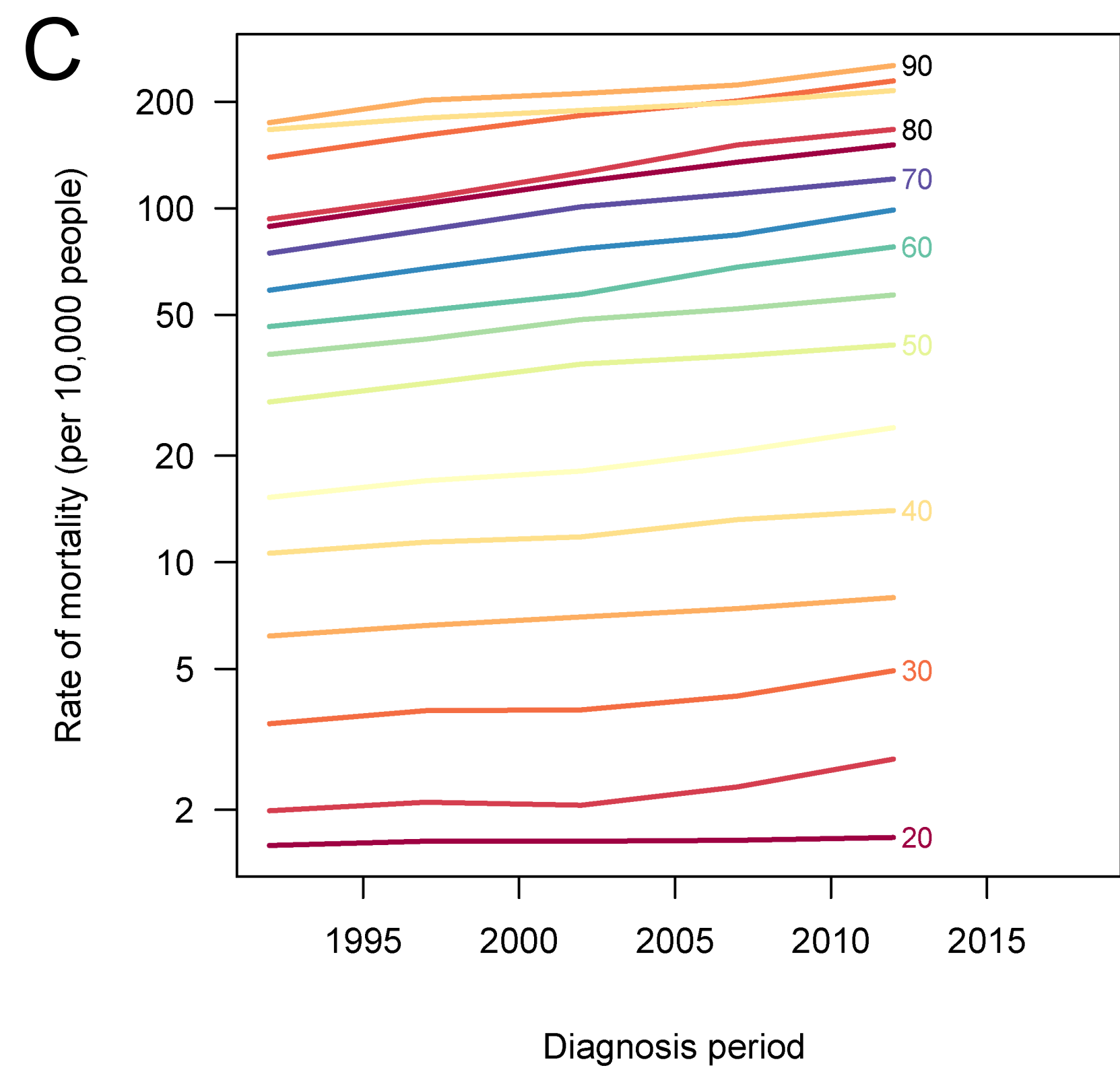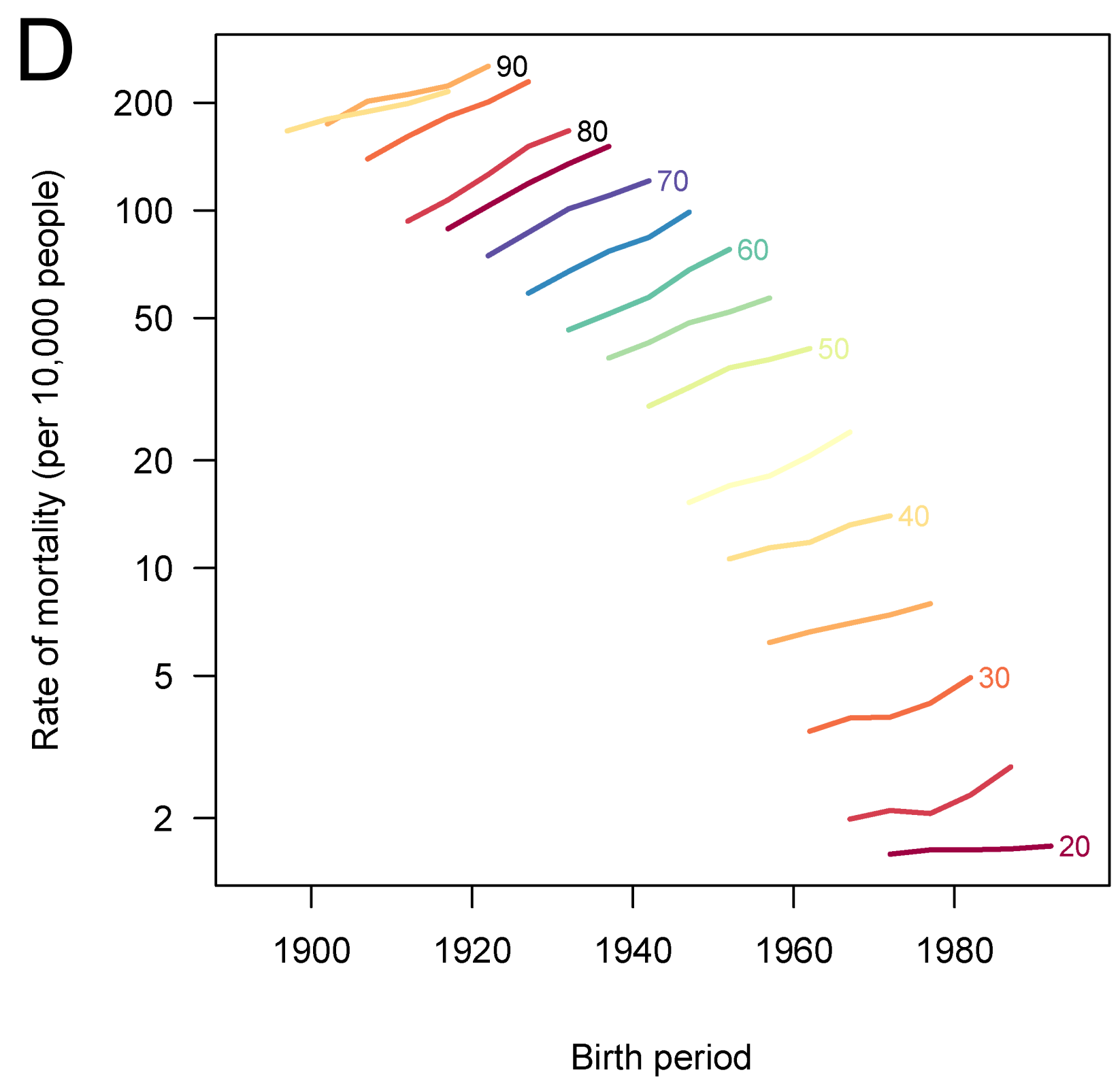

Supplement: Figure S3 — Age-period-cohort analysis of mortality rates for total cancer attributable to high BMI in China, 1990-2021. (A) The age-specific mortality rates according to calendar periods; each line connects the mortality rates for a given 5-year period. (B) The age-specific mortality rates according to birth cohorts; each line connects the mortality rates for a given 5-year birth cohort. (C) The period-specific mortality rates according to age groups; each line connects the mortality rates for a given 5-year age group. (D) The cohort-specific mortality rates according to age groups; each line connects the mortality rates for a given 5-year age group. BMI, body mass index. [file Image_3.pdf]
