# Supplementary material for: Long-term trends in the burden of cancer attributable to high body mass index in China from 1990 to 2021
Source: Front Nutr. 2025 May 21;12:1606747. doi: 10.3389/fnut.2025.1606747 (PMC12133465; doi:10.3389/fnut.2025.1606747)
Supplement: Figure S2 — Sex distribution of site-specific cancer burden attributable to high BMI in China in 2021. (A) Sex distribution by cause for deaths. (B) Sex distribution by cause for DALYs. (C) Sex distribution by cause for YLLs. (D) Sex distribution by cause for YLDs. DALYs, disability-adjusted life years; YLDs, years lived with disability; YLLs, years of life lost; BMI, body mass index. [file Image_2.pdf]

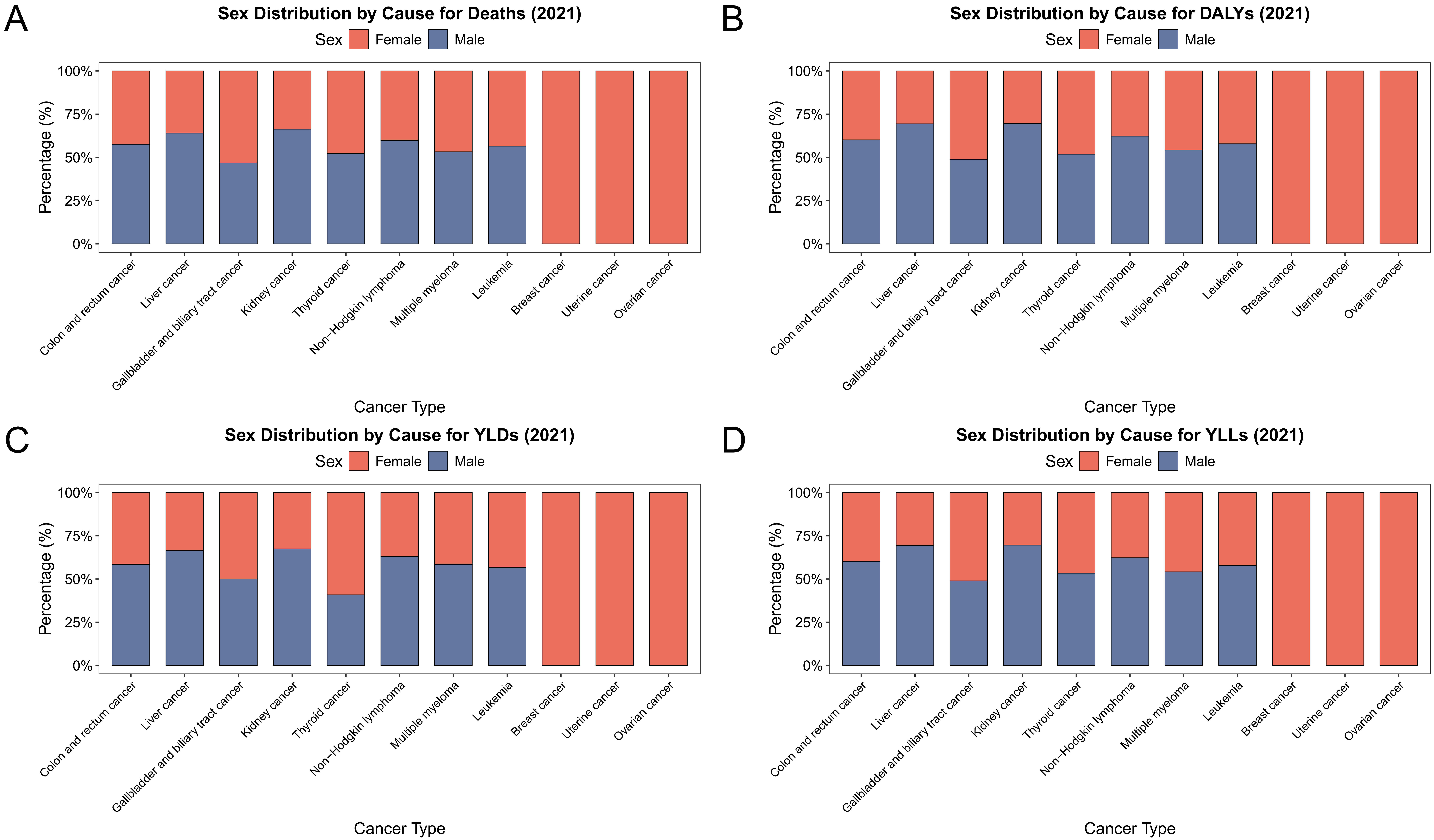
A
Sex Distribution by Cause for Deaths (2021)
B
Sex Distribution by Cause for DALYs (2021)
C
Sex Distribution by Cause for YLDs (2021)
D
Sex Distribution by Cause for YLLs (2021)
Sex
Female
Male
Percentage (%)
100%
75%
50%
25%
0%
Colon and rectum cancer
Liver cancer
Gallbladder and biliary tract cancer
Kidney cancer
Thyroid cancer
Non−Hodgkin lymphoma
Multiple myeloma
Leukemia
Breast cancer
Uterine cancer
Ovarian cancer
Cancer Type
