# Supplementary material for: Long-term trends in the burden of cancer attributable to high body mass index in China from 1990 to 2021
Source: Front Nutr. 2025 May 21;12:1606747. doi: 10.3389/fnut.2025.1606747 (PMC12133465; doi:10.3389/fnut.2025.1606747)
Supplement: Supplementary file 4 [file Table_1.docx]

Table S1. YLDs and YLLs for total cancer attributable to high BMI in China, 2021, with trends in ASRs per 100,000 population, 1990 - 2021

|  | YLDs | | | YLLs | | |
| --- | --- | --- | --- | --- | --- | --- |
| Cancer type | No, in thousands | Age-standardized rate per 100,000 | Percentage change from 1990 to 2021 | No, in thousands | Age-standardized rate per 100,000 | Percentage change from 1990 to 2021 |
| Total cancers | 71.3 (25.9, 123.8) | 3.3 (1.2, 5.7) | 303.1 (206.8, 411.6) | 1587.4 (667.4, 2709) | 75.9 (32.6, 128.5) | 98 (46, 154.4) |
| Colon and rectum cancer | 24.4 (9.2, 41.7) | 1.1 (0.4, 2) | 375.3 (264.1, 561.7) | 482.9 (200.4, 815.8) | 23.1 (9.6, 38.9) | 98.3 (50.4, 180.1) |
| Liver cancer | 3.5 (1.3, 6.5) | 0.2 (0.1, 0.3) | 270 (158.6, 400.4) | 375.8 (147.5, 690.2) | 18.1 (7.1, 33.2) | 192 (103.3, 300) |
| Gallbladder and biliary tract cancer | 1.5 (0.8, 2.4) | 0.1 (0, 0.1) | 129 (67.7, 204.5) | 94.1 (53.1, 140.7) | 4.4 (2.5, 6.6) | 40.9 (1.8, 86.8) |
| Pancreatic cancer | 0 (-0.3, 0.6) | 0 (0, 0) | -109.9 (-438.7, -4.5) | 7.6 (-32.4, 75.9) | 0.3 (-1.5, 3.5) | -115.1 (-437.5, -13.6) |
| Kidney cancer | 4.2 (1.6, 7.5) | 0.2 (0.1, 0.4) | 438.9 (304.8, 594.7) | 92.5 (34.3, 162.5) | 4.4 (1.6, 7.8) | 156.2 (93.7, 230.1) |
| Thyroid cancer | 2.9 (1.8, 4.6) | 0.1 (0.1, 0.2) | 231.1 (145.9, 346.4) | 20.8 (13.9, 28.1) | 1 (0.7, 1.4) | 16 (-9.2, 51.9) |
| Non-Hodgkin lymphoma | 3 (0.9, 5.6) | 0.1 (0, 0.3) | 603.3 (424.7, 827.9) | 48.9 (15.6, 86.6) | 2.4 (0.8, 4.2) | 51.4 (11, 97) |
| Multiple myeloma | 0.6 (-0.2, 1.5) | 0 (0, 0.1) | 1167.8 (383, 3486.3) | 18.6 (-6.3, 47.7) | 0.9 (-0.3, 2.3) | 632.3 (170, 2005.5) |
| Leukemia | 4 (2.3, 6.2) | 0.2 (0.1, 0.3) | 120.9 (69.3, 193) | 140 (94.1, 193) | 7.6 (5.1, 10.5) | 4.6 (-18.6, 33.6) |
| Breast cancer | 15.8 (-0.5, 32.9) | 0.7 (0, 1.4) | 322.8 (208.6, 461.7) | 153.6 (-5, 332.8) | 6.6 (-0.2, 14.3) | 94.7 (34.7, 172.5) |
| Uterine cancer | 9.9 (5.2, 16.3) | 0.5 (0.2, 0.7) | 195.2 (105.5, 333.9) | 101.1 (60.9, 157.4) | 4.7 (2.8, 7.3) | 16.6 (-18.1, 68) |
| Ovarian cancer | 1.6 (0.3, 3.3) | 0.1 (0, 0.2) | 555 (-2580.4, 3064.4) | 51.4 (10.1, 105.7) | 2.4 (0.5, 4.8) | 406.3 (-1790, 2813.4) |

Values in parentheses indicate 95% UIs, estimated using Monte Carlo simulations. Extreme percentage changes may occur when baseline ASRs in 1990 are close to zero. Abbreviations: YLDs, years lived with disability; YLLs, years of life lost; BMI, body mass index; ASRs, Age-standardized rates; UI, uncertainty interval.
