# Supplementary material for: Long-term trends in the burden of cancer attributable to high body mass index in China from 1990 to 2021
Source: Front Nutr. 2025 May 21;12:1606747. doi: 10.3389/fnut.2025.1606747 (PMC12133465; doi:10.3389/fnut.2025.1606747)
Supplement: Supplementary file 5 [file Table_2.docx]

Table S2. Trends in age-standardized mortality, DALY, YLD, and YLL rates (per 100,000 persons) among both sexes, males, and females from 1990 to 2021 for total cancer attributable to high BMI in China.

|  | Age-standardized mortality rate | | | Age-standardized DALY rate | | | Age-standardized YLD rate | | | Age-standardized YLL rate | | |
| --- | --- | --- | --- | --- | --- | --- | --- | --- | --- | --- | --- | --- |
| Gender | Period | APC (95% CI) | AAPC (95% CI) | Period | APC (95% CI) | AAPC (95% CI) | Period | APC (95% CI) | AAPC (95% CI) | Period | APC (95% CI) | AAPC (95% CI) |
| Both | 1990-1997 | 1.77 (1.56 - 1.94) ^*^ | 2.40 (2.37 - 2.44) ^*^ | 1990-1997 | 1.60 (1.37 - 1.77) ^*^ | 2.33 (2.30 - 2.36) ^*^ | 1990-1996 | 2.95 (2.63 - 3.17) ^*^ | 4.62 (4.58 - 4.66) ^*^ | 1990-1997 | 1.57 (1.33 - 1.73) ^*^ | 2.25 (2.22 - 2.28) ^*^ |
|  | 1997-2001 | 3.39 (2.86 - 3.94) ^*^ |  | 1997-2001 | 3.12 (1.87 - 3.59) ^*^ |  | 1996-2002 | 5.02 (4.12 - 5.31) ^*^ |  | 1997-2001 | 3.07 (2.49 - 3.55) ^*^ |  |
|  | 2001-2007 | 1.63 (1.23 - 1.91) ^*^ |  | 2001-2007 | 1.59 (1.20 - 3.07) ^*^ |  | 2002-2010 | 5.79 (5.40 - 6.08) ^*^ |  | 2001-2007 | 1.46 (1.07 - 1.76) ^*^ |  |
|  | 2007-2010 | 3.01 (2.33 - 3.33) ^*^ |  | 2007-2010 | 2.92 (1.60 - 3.23) ^*^ |  | 2010-2016 | 3.70 (3.39 - 5.82) ^*^ |  | 2007-2010 | 2.91 (1.71 - 3.19) ^*^ |  |
|  | 2010-2014 | 1.91 (1.42 - 2.38) ^*^ |  | 2010-2014 | 2.00 (1.55 - 2.51) ^*^ |  | 2016-2019 | 6.17 (3.64 - 6.56) ^*^ |  | 2010-2013 | 1.64 (1.38 - 2.35) ^*^ |  |
|  | 2014-2021 | 3.17 (2.99 - 3.44) ^*^ |  | 2014-2021 | 3.19 (3.01 - 3.50) ^*^ |  | 2019-2021 | 4.27 (3.49 - 5.47) ^*^ |  | 2013-2021 | 3.03 (2.89 - 3.27) ^*^ |  |
| Female | 1990-1997 | 1.62 (1.18 - 1.83) ^*^ | 1.98 (1.93 - 2.02) ^*^ | 1990-1997 | 1.38 (0.92 - 1.58) ^*^ | 1.82 (1.77 - 1.86) ^*^ | 1990-1996 | 3.10 (2.67 - 3.36) ^*^ | 4.27 (4.23 - 4.31) ^*^ | 1990-1997 | 1.33 (0.93 - 1.52) ^*^ | 1.73 (1.68 - 1.76) ^*^ |
|  | 1997-2001 | 2.77 (2.25 - 3.34) ^*^ |  | 1997-2001 | 2.60 (2.09 - 3.14) ^*^ |  | 1996-2002 | 4.61 (3.93 - 4.97) ^*^ |  | 1997-2001 | 2.50 (2.02 - 3.03) ^*^ |  |
|  | 2001-2015 | 1.30 (1.19 - 1.38) ^*^ |  | 2001-2015 | 1.17 (1.06 - 1.25) ^*^ |  | 2002-2007 | 5.70 (5.34 - 6.35) ^*^ |  | 2001-2015 | 1.04 (0.95 - 1.12) ^*^ |  |
|  | 2015-2021 | 3.47 (3.12 - 3.93) ^*^ |  | 2015-2021 | 3.34 (3.04 - 3.81) ^*^ |  | 2007-2011 | 3.99 (3.44 - 4.57) ^*^ |  | 2015-2021 | 3.27 (2.98 - 3.69) ^*^ |  |
|  |  |  |  |  |  |  | 2011-2015 | 2.29 (1.64 - 2.76) ^*^ |  |  |  |  |
|  |  |  |  |  |  |  | 2015-2021 | 5.45 (5.17 - 5.78) ^*^ |  |  |  |  |
| Male | 1990-1997 | 2.02 (1.81 - 2.19) ^*^ | 2.94 (2.90 - 2.97) ^*^ | 1990-1997 | 1.89 (1.69 - 2.06) ^*^ | 2.88 (2.83 - 2.91) ^*^ | 1990-1995 | 2.78 (2.07 - 3.09) ^*^ | 5.30 (5.26 - 5.34) ^*^ | 1990-1997 | 1.87 (1.66 - 2.04) ^*^ | 2.82 (2.78 - 2.86) ^*^ |
|  | 1997-2001 | 4.29 (3.87 - 4.91) ^*^ |  | 1997-2001 | 3.90 (3.51 - 4.49) ^*^ |  | 1995-1998 | 4.17 (3.31 - 6.23) ^*^ |  | 1997-2001 | 3.87 (3.47 - 4.45) ^*^ |  |
|  | 2001-2007 | 2.22 (1.81 - 2.45) ^*^ |  | 2001-2006 | 1.94 (1.46 - 2.24) ^*^ |  | 1998-2006 | 6.29 (5.97 - 7.11) ^*^ |  | 2001-2006 | 1.84 (1.36 - 2.15) ^*^ |  |
|  | 2007-2010 | 4.43 (3.75 - 4.76) ^*^ |  | 2006-2015 | 3.69 (3.56 - 3.95) ^*^ |  | 2006-2012 | 7.09 (5.14 - 7.63) ^*^ |  | 2006-2015 | 3.62 (3.49 - 3.88) ^*^ |  |
|  | 2010-2021 | 3.03 (2.89 - 3.11) ^*^ |  | 2015-2021 | 2.92 (2.48 - 3.16) ^*^ |  | 2012-2021 | 5.05 (4.86 - 5.23) ^*^ |  | 2015-2021 | 2.86 (2.42 - 3.11) ^*^ |  |

Abbreviations: DALYs, disability-adjusted life-years; YLDs, years lived with disability; YLLs, years of life lost; BMI, body mass index; AAPC, average annual percent change presented for full period; APC, annual percent change; CI, confidence interval. ^*^, *p* < 0.05.
